# Sources and Extraction of Biopolymers and Manufacturing of Bio-Based Nanocomposites for Different Applications

**DOI:** 10.3390/molecules29184406

**Published:** 2024-09-16

**Authors:** Elham Azadi, Mohammad Dinari, Maryam Derakhshani, Katelyn R. Reid, Benson Karimi

**Affiliations:** 1 Department of Chemistry, Isfahan University of Technology, Isfahan 84156-83111, Iran; elhamazadi1366@yahoo.com (E.A.);; 2 Department of Physical and Environmental Sciences, Texas A&M University Corpus Christi, Corpus Christi, TX 78412, USA

**Keywords:** green sources, extraction, polysaccharides, bio-nanocomposites, applications

## Abstract

In the recent era, bio-nanocomposites represent an emerging group of nanostructured hybrid materials and have been included in a new field at the frontier of materials science, life sciences, and nanotechnology. These biohybrid materials reveal developed structural and functional features of great attention for diverse uses. These materials take advantage of the synergistic assembling of biopolymers with nanometer-sized reinforcements. Conversely, polysaccharides have received great attention due to their several biological properties like antimicrobial and antioxidant performance. They mainly originated in different parts of plants, animals, seaweed, and microorganisms (bacteria, fungi, and yeasts). Polysaccharide-based nanocomposites have great features, like developed physical, structural, and functional features; affordability; biodegradability; and biocompatibility. These bio-based nanocomposites have been applied in biomedical, water treatment, food industries, etc. This paper will focus on the very recent trends in bio-nanocomposite based on polysaccharides for diverse applications. Sources and extraction methods of polysaccharides and preparation methods of their nanocomposites will be discussed.

## 1. Introduction

Annually, in producing plastic-based materials, 40% of global oil production is used. For example, around the world, one million plastic bags are used every minute. Additionally, with the emergence of the COVID-19 pandemic, plastic consumption has increased to more than 8 million tons. Most conventional plastics, including polyethylene, polypropylene, polystyrene, poly(vinyl chloride), and poly(ethylene terephthalate), are non-biodegradable, and their increasing accumulation in the environment leads to contamination in ecosystems (living beings, waterways, oceans) and health hazards for humans. Therefore, new alternatives with green properties to replace these synthetic plastics have excessive significance [1,2,3,4].

Presently, there is a rising affinity for employing eco-friendly resources to substitute non-degradable materials and decrease the environmental pollution from plastics. Indeed, in recent years, petroleum-derived synthetic plastics have been replaced with biopolymers [1]. The term biopolymer originates from the Greek words bio and polymer, representing nature and living organisms (such as plants, animals, microorganisms, and agricultural wastes). Biopolymers are classified based on their origin. For example, synthetic biopolymers like polylactic acid (PLA) and polyhydroxyalkanoates (PHAs) are two examples of biopolymers found in microorganisms or genetically modified organisms utilizing traditional chemical methods. However, the most commonly employed and examined biopolymers are natural biopolymers like chitosan, alginate, starch, pectin, and cellulose [4]. In addition to the renewability, availability, and eco-friendly nature of these biopolymers, some of them have shown antioxidant, antifungal, and antimicrobial performance, so they are employed in numerous industries [2,5,6,7,8,9,10,11]. Polysaccharides predominantly originate in different parts of plants, animals, seaweed, mushrooms, and microorganisms (bacteria, fungi, and yeasts) [12] and play an important role in various physiological purposes of life, like as components of the human diet [13,14]. In fact, dietary polysaccharides have crucial influences on human health [15]. Biological benefits for bowel health, including anti-inflammation, gut epithelial barrier protection, and immune modulation through both microbiota-dependent and -independent mechanisms. Recently, interest in the role of polysaccharides in preventing obesity and non-alcoholic fatty liver disease has increased [12].

Until now, polysaccharides have been extracted from various sources. For example: Flórez-Fernández et al. reported ultrasound-assisted aqueous extraction of alginate from *Sargassum muticum* (seaweeds) [16]. Benslima et al. used brown seaweed *Cystoseira schiffneri* as a source of alginate, and the prepared biopolymer showed good antioxidant action [17]. Kaya et al. extracted chitin/chitosan with antioxidant as well as antimicrobial performance from cosmopolitan Orthoptera *species* (Insecta) [18]. Uğurlu et al. reported the extraction of chitin and chitosan from the invasive sea urchin Diadema *setosum* [19]. In another study by Tissera et al., high-quality chitosan was obtained from blue swimmer (*Portunus pelagicus*) crab shell waste [20]. Spinei et al. examined the extraction of pectin from grape pomace by microwave method [21]. Also in an investigation by Liew et al., passion fruit peels were used to extract pectin [22].

Bio-based nanocomposites are hybrid materials composed of naturally occurring polymers in combination with nano-dimensional material. There are four types of nano-fillers: clays, organic, inorganic, and carbon nanostructure. Due to the outstanding properties of these nano-hybrids as structural or functional materials, materials scientists have made huge efforts in this field [5,23,24]. Bio-nanocomposites have the outstanding benefits of exhibiting environmentally friendly, improved physical, structural, and functional features, affordable, biodegradability, non-toxicity, and biocompatibility [2,25]. Nano-reinforcements in composites deliver remarkably improved properties, such as a decrease in hydrophilicity and an increase in mechanical performance. So far, diverse applications of bio-nanocomposites have been reported including biomedicine, building, agriculture, textile, food, electrochemical devices, catalysts, and water treatment [26,27,28,29,30,31,32,33]. Bio-nanocomposite based on these polysaccharides can be synthesized in several ways, like film casting, electrospinning, co-precipitation, in situ preparation, and dip coating [24]. Until now, diverse bio-nanocomposites based on polysaccharides have been developed for numerous applications. For example, Khorasani et al. manufactured pectin-based bio-nanocomposites as prebiotics against drying and gastrointestinal conditions [34]. Govindaraj and co-workers prepared pectin/apatite bio-nanocomposites derived from Jackfruit peel for bone healing [35]. Satriaji et al. designed antibacterial bio-nanocomposite films for food packaging based on CaSO_4_-crosslinked alginate/ZnO nanoparticles [36]. Ahmad and Ansari fabricated of bio-nanocomposite of alginate@Ag for the sequestration of crystal violet from water [37]. Wei et al. reported the successful adsorption of arsenate from water by activated MOF-embedded chitosan bio-nanocomposite beads [38]. Christy P. and co-workers reported an antibacterial hybrid bio-nanocomposite of chitosan/poly(vinyl alcohol)/nano-bioactive glass/nano-cellulose for bone tissue engineering [39]. Due to the reduction of fossil fuels and the increase in the pollution of synthetic plastics, in recent years, much attention has been paid to natural polymers’ special polysaccharides. Many research works and review articles and books have been published in this regard. Based on the importance of polysaccharides and their nanocomposites, this review will focus on many very common polysaccharides, their sources, their extraction methods, and very recent trends in preparation methods of the bio-nanocomposites for diverse applications, including food packaging and water treatment, will be explained. This paper will provide a good reference for researchers who work in many industrial fields like packaging.

## 2. Alginate

**Figure 1:**
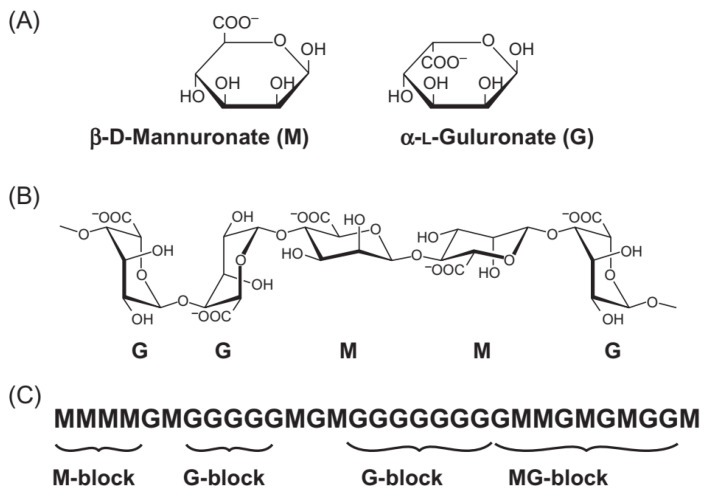
Structural characteristics of alginates: (**A**) Alginate monomers, (**B**) chain conformation, and (**C**) block distribution. Reproduced with permission from [51], 2021, Elsevier.

As an anionic straight-chain polysaccharide, alginate with the (C_6_H_7_NaO_6_)_n_ molecular formula is collected from repeating units of α-L-guluronic acid (G) residues and β-D-mannuronic acid (M) parts linked via 1–4 glycosidic connections as shown in Figure 1. Alginate molecules can be in three following fractions: manuronics (MMM), glucuronics (GGG), as well as manuronic/glucuronics mixture (MGM) [40,41,42,43]. Many important factors contribute to the sequence and composition of M and G parts that influence on different alginate properties including natural resources, season of the year, location, extraction tissue for alginate, and age of the tissue [41,42]. The structural chain of alginate is rich in carboxyl and hydroxyl functional groups. In comparison with many other polysaccharides like gelatin, alginate can form gels independently of temperature changes. Indeed, alginic acids form water-soluble salts with monovalent cations like sodium but are precipitated in the presence of polyvalent cations. The gel formation is reached by ionic bonding with cations after exposure to a salt environment. In the presence of cations such as Ca^2+^ (CaCl_2_ solution), an interaction (strong ionic bonds) can occur among COO^−^ groups of guluronic acids and Ca^2+^ ions, and a 3D lattice insoluble that is referred to as the egg box can be created. Alginate expresses different affinity towards various cations to form gels (i.e., Pb^2+^ > Cu^2+^ > Cd^2+^ > Ba^2+^ > Sr^2+^ > Ca^2+^) [42,44].

Alginate, this linear, eco-friendly, non-toxic, water-soluble, pH-sensitive, biodegradable, perishable, nonimmunogenic, biocompatible, and poly-anionic polymer, is obtained from the algae cell wall and various species of bacteria [42]. This abundant polysaccharide extracted from brown algae has commercial importance. In 1881, sodium alginate was extracted from seaweed by the ion exchange technique and described by the British chemist E. C. C. Stanford, and its patent was released in the market [41,42]. In the late 1920s, alginate was produced on a commercial level. Due to its countless benefits, such as the capability to retain water, low cost, gel formation, viscosity, mucoadhesivity, and stabilizing features, it is extensively used in industry. Commercially available native alginate has a molecular weight ranging between 32,000 and 400,000 g/mol. Many significant features of alginates similar gel formation and viscosity are dependent on the Mw. Alginate with high Mw can produce a very viscous gel, which is not practicable for industrial uses [40,41,42,43]. Alginate is extensively employed in numerous applications like the health field, electronics, food, pharmaceutical, drug delivery, tissue engineering, dental impression material, wound healing, textiles, food, agri-foods, cosmetics, and paper [40,41,45,46,47,48].

### 2.1. Sources and Extraction of Alginate

Alginates have two main sources: seaweed (the cell wall of brown marine algae like *Phaeophyceae* family) and bacteria (like *Azotobacter* and *Pseudomonas*). Commercial ones are extracted from algae sources though microbial fermentation is feasible for manufacture. Many sources of brown marine algae are *Ascophyllum nodosum*, *Laminaria hyperborea*, and *Macrocystis pyrifera* [49,50,51].

**Figure 2:**
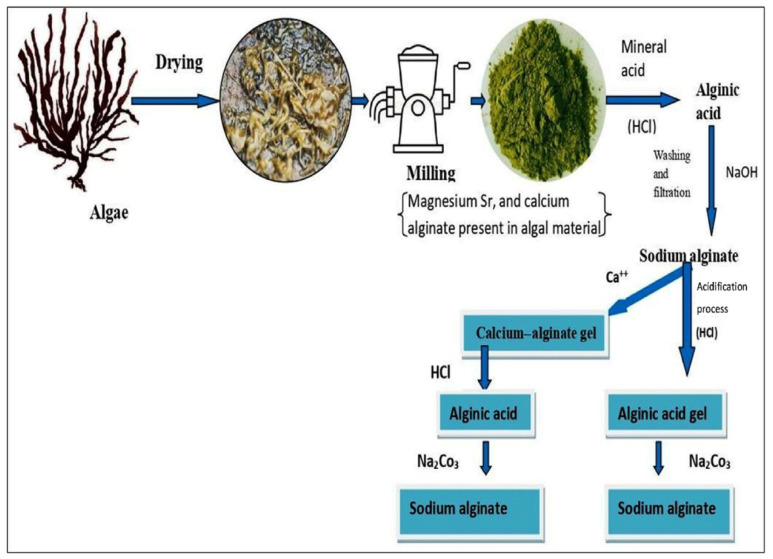
The schematic flowchart for the extraction of alginates from the algal sources. Reproduced with permission from [49], 2020, Elsevier.

The general procedure for extraction of alginates from brown algae has multiple steps. At first, raw algae are mechanically harvested and then dried (the exceptional candidate is *M. pyrifera* used in wet conditions). After that, the dried materials are treated with mineral acids (for the elimination of counter ions via the exchanging of proton). Next, through solubilizing insoluble fractions of alginic acid by neutralization process (an alkali agent similar to NaOH or Na_2_CO_3_ is used), alginate is extracted. To separate the precipitates, centrifugation or floatation is carried out. The precipitations are treated with alcohols or mineral acids to directly extract sodium (Na)-alginate. This step is due to eliminating impurities (degrading the homopolysaccharides, for example, fucoidin and laminarin). An alternative procedure to extract alginate is using Ba or Ca ions to produce stable alginate gel. Lastly, pure Na-alginate is extracted through treating by alkaline solutions (Figure 2) [49,50,51].

Well-known bacterial sources for the preparation of alginate are *Azotobacter* sp. and *Pseudomonas* sp. pathogens. The pathway of bacterial synthesis includes (1) production of precursor substrate, (2) polymerization and cytoplasmic membrane transfer, (3) periplasmic transfer and modification, and (4) export through the outer membrane. Until now, this polymer was extracted from *Sargassum cristaefolium* [52], *Sargassum biomass* [53,54], *Sargassum turbinarioides* [55], various *species* from Fucales order (Phaeophyceae) [56], *Sargassum angustifolium*, etc. [57]. In the following, some new studies on the extraction of alginate from seaweed and bacterial sources are mentioned and summarized in Table 1 [49].

**Figure 3:**
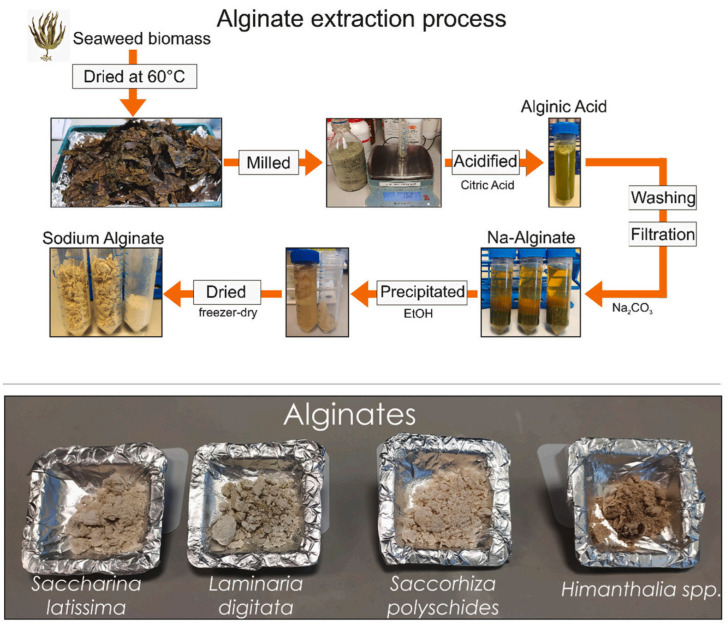
Alginate extraction from Saccharina latissima, Laminaria digitata, Sacchoriza polyschides, and Himanthalia spp. Reproduced from [58], 2023, Elsevier, Available under CC BY license.

In 2023, Silva et al. extracted commercial alginate from different *species* of brown algae of *Saccharina latissima* (SAC), *Laminaria digitata* (LAM), *Sacchoriza polyschides* (SACC), and *Himanthalia* spp. (HIM) [58]. For this aim, 3% *w/v* of milled seaweed biomass was acidified using citric acid solution (4%) in 200 rpm shaking at 30 °C for 12 h. Then, the biomass was filtered and washed with water. After collection, it was re-suspended in Na_2_CO_3_ solution and shaken for 24 h. By centrifugation (3500 rpm), soluble fractions were collected. Finally, Na-alginate was precipitated with absolute ethanol, filtered, and dried (Figure 3). After extraction, crude alginate represented 61–65% of the biomass dry weight for SAC and LAM and 34–41% for SACC and HIM when experiments were performed at a small scale (1.5 g of starting material). Remarkably, scaling up extraction (60 g of starting material) decreased yields to 26–30%. The extracted alginates from *Saccharina latissima*, and *Laminaria Digitata* showed the highest Mw (302 and 362 kDa) and mannuronic/guluronic ratio (M/G: 1.98 and 2.23). The obtained alginates were tested for spinning fibers. The outcomes showed the production of fibers only by SAC and LAM without clumps or cracks with 2.4 and 2.0 GPa Young’s modulus. Fibers produced from LAM alginates showed a linear response, followed by plastic deformation and net failure at 11.41 % elongation. Similarly, SAC fibers initially showed rigid response and then plastic deformation leading to failure at 9.47% elongation.

**Figure 4:**
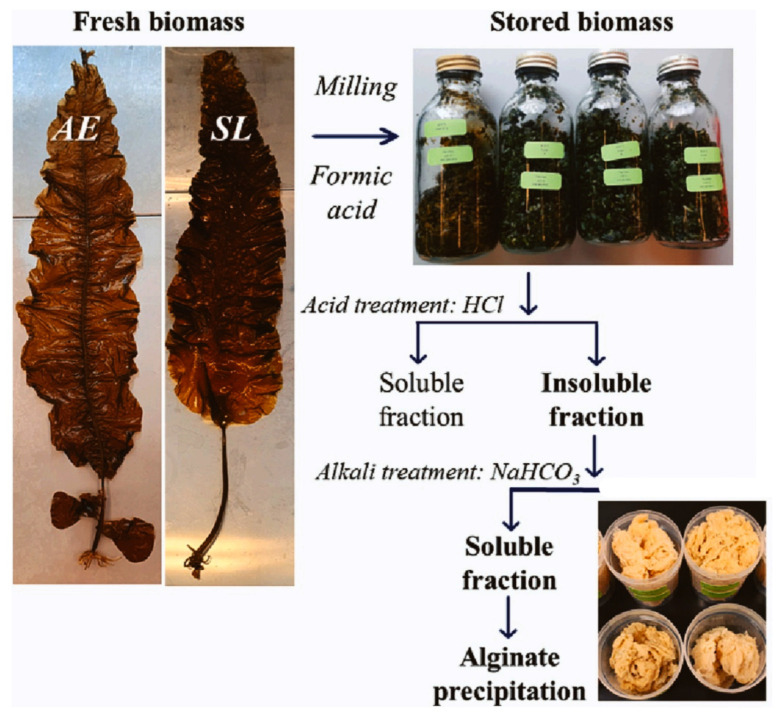
Processing scheme for acid preservation of *Alaria esculenta* (AE) and *Saccharina latissima* (SL) and subsequent alginate extraction [59,60]. Adapted from [60], 2023, Elsevier, Available under CC BY license.

Presently, Norway is the largest manufacturer of cultivated seaweed in Europe, where the major *species* are Saccharina latissima and Alaria esculenta. So, Nøkling-Eide et al. extracted alginate from two brown algal *species* [59,60] (*Alaria esculenta* and *Saccharina latissima*) in Norway. Before extraction and characterization of alginate, algal *species* were preserved with formic acid at 4, 13, and 20 °C for up to 16 weeks. For the extraction of alginate, the dried biomass was added into HCl (0.2 M) and incubated over (20 °C) (Figure 4). After centrifuging and washing the biomass, NaHCO_3_ was added. (pH: 7.2–7.5). After decant and filtration, solid residues were lyophilized. Finally, the alginate was centrifuged and washed. The results showed an increased yield (up to 40%) of alginate from preserved biomass compared to the non-preserved biomass.

**Figure 5:**
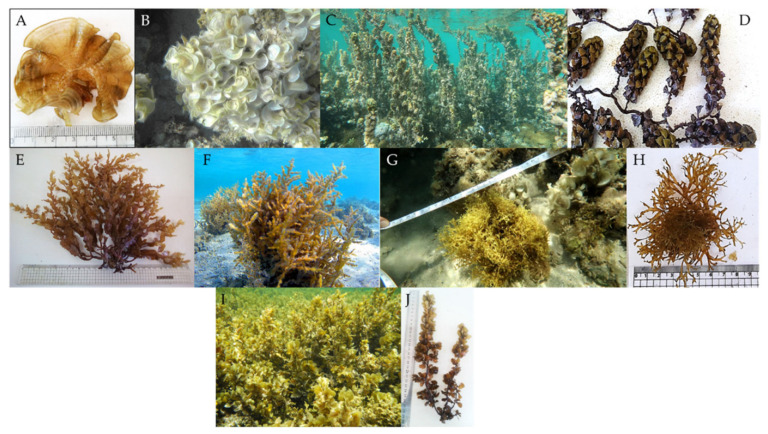
Seaweed *species* images: *Padina boergesenii*: (**A**) whole thallus of the *species* and (**B**) view in the natural habitat at the NIOF (National Institute of Oceanography and Fisheries) site. *Turbinaria triquetra:* (**C**) view in the natural habitat at the NIOF site and (**D**) whole thallus of the species. *Hormophysa cuneiformis:* (**E**) whole thallus of the species and (**F**) view in the natural habitat at the NIOF site. *Dictyota ciliolate* (**G**) view in the natural habitat at the NIOF site and (**H**) whole thallus of the species. *Sargassum aquifolium* (**I**) view in the natural habitat at the NIOF site and (**J**) whole thallus of the species. Reproduced from [61], 2021, MDPI, Available under CC BY license.

In another work by Rashedy et al., different *species* of dominant brown seaweeds (*Padina boergesenii*, *Turbinaria triquetra*, *Hormophysa cuneiformis*, *Dictyota ciliolata*, and *Sargassum aquifolium*) (Figure 5) were obtained in the Red Sea to extract alginate [61]. For the extraction, powdered seaweeds were immersed in CH_2_O for one day. Next, they were filtered and washed with deionized water. After adding 0.2 M HCl solution, they were filtered and washed. Then, Na_2_CO_3_ was added to the residue. Alginate was extracted and filtered. The filtrate was bleached with NaClO. Finally, the precipitated sodium alginate was obtained using ethanol and then washed and dried. Based on the outcomes, *H. cuneiformis* (13.3 *±* 0.52% DW) and *T. triquetra* (22.2 *±* 0.56% DW) showed the highest and lowest yields of product. The moisture content of alginate was 6.4–13.1%. The produced alginate from all of the samples showed food-grade quality when compared to the alginate international regulation.

In the following, some alginate-based bio-nanocomposites for diverse applications that have been published in 2023 will be mentioned and summarized in Table 2.

### 2.2. Alginate-Based Nanocomposites

**Figure 6:**
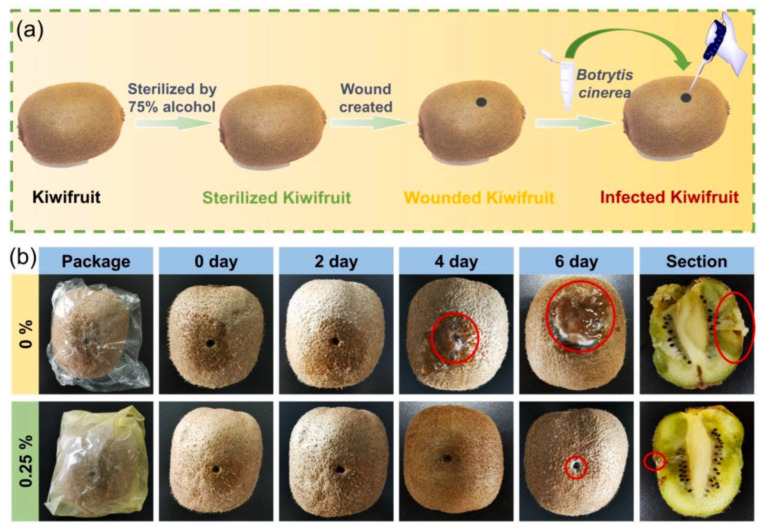
(**a**) Schematic illustration of fungal infection in kiwifruit; (**b**) inhibitory effect of chitosan/alginate/quantum dots @ZIF-8 nanocomposite films on the surface mold of kiwifruit. Reproduced with permission from [62], 2023, Elsevier.

For the preservation of vegetables/fruits, packaging technologies are used owing to their widespread applicability and versatility in addition to ease of handling. Especially, the creation of several types of green food packaging based on polysaccharides not only efficiently avoids the potential safety hazards and white pollution of synthetic petroleum-based plastics but is also easy to functionalize because of its safe source. In this regard, Wang et al. fabricated visible-light-responsive nanocomposite films based on Na-alginate, chitosan, quantum dots, and metal–organic frameworks (ZIF-8) [62]. For this aim, first, quantum dots/ZIF-8 nanoparticles as nanofillers were prepared: Zn–Ag–In–S quantum dots were fabricated in a hydrothermal process. By embedding the prepared quantum dots/ZIF-8 nanofiller uniformly in the chitosan/alginate matrix via the layer-by-layer (LBL) assembly method, nanocomposite films were prepared. In this work, three present quantum dots/ZIF-8 nanoparticles (0.1%, 0.25%, and 0.5%) were dispersed in the matrix. For crosslinking the materials, CaCl_2_ and trisodium citrate solution were used. Different analyses, including FT-IR, XRD, XPS, UV–Vis, and SEM-EDX, indicated the successful fabrication of the nanocomposite films. After analyzing the appearance/color of films, they showed high transparency, while the transparency of the films slowly declined with the increase in quantum dots/ZIF-8 nanoparticles. Thermal stability, as a significant indicator of the nanocomposite performance, was determined via thermo-gravimetric analysis in 25–600 °C decomposition temperature. The outcomes showed two processes of weight loss: 30–220 °C due to water evaporation, and 220–300 °C due to Na-alginate and chitosan degradation, and eventually the weight loss of each group of composite films flattens out, maybe due to the presence of non-combustible minerals in the films. Additionally, mechanical properties (tensile strength and elongation at break) were tested. The tensile strength of films increased from 25.5 MPa to 28.6 MPa, 30.3 MPa, and 27.8 MPa for 0.1%, 0.25%, and 0.5% nanocomposite films, respectively. The significant increase in the tensile strength value of films was attributed to the strong -H bonding interaction among polymeric matrix and well-dispersed quantum dots/ZIF-8 nanoparticles. In the next step, antimicrobial and ethylene scavenging action to preserve kiwifruit was tested. The fabricated nanocomposite film showed >99% antibacterial performance against foodborne microorganisms under visible light irradiation and >40% ethylene scavenging feature owing to the synergism of reactive oxygen species (ROS) as well as the high specific surface area of MOF (metal–organic frameworks). Additionally, these nanocomposite films displayed important enhancement in thermal stability, mechanical, thermal, hydrophobicity, and barrier capacity by incorporation of quantum dots/ZIF-8, which proved the feasibility of their application in kiwifruit preservation. Furthermore, nanocomposites’ biosafety was demonstrated. For testing fungal infection in kiwifruit, at first, the desired fruit was sterilized with alcohol, and a wound (3 mm diameter) was created in it. *B. cinerea* was injected into it, and kiwifruit was wrapped in the prepared nanocomposite film. By using a light source, the wound area was irradiated and kept at room temperature. The inhibitory effect of nanocomposite films on the kiwifruit surface can be seen in Figure 6. In the absence of nanocomposite, molds started to grow from the 4th day onwards.

**Figure 7:**
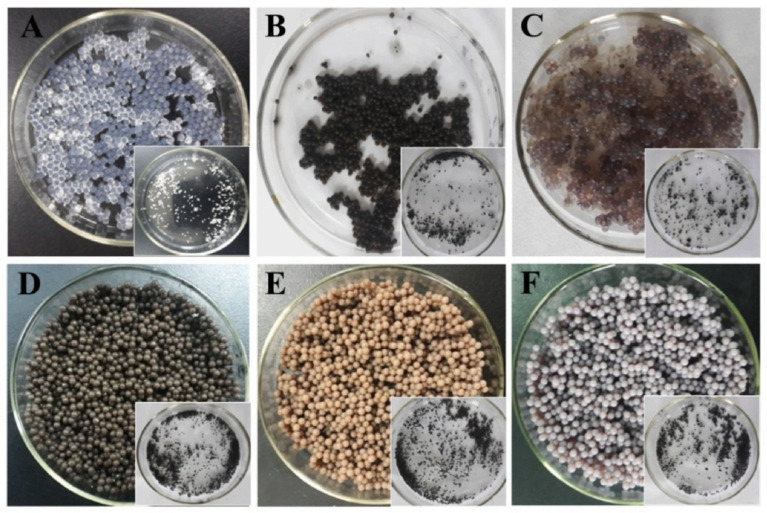
Silver nanocomposite micro-gel beads, (**A**) plain calcium alginate, (**B**) adsorption–reduction method, (**C**) nanosilver particles adsorption method, (**D**) incorporation of preparing NSPs in the calcium alginate method, (**E**) incorporation of NSPs in calcium alginate method, and (**F**) gelation–reduction method. Reproduced with permission from [63], 2023, Elsevier.

**Figure 8:**
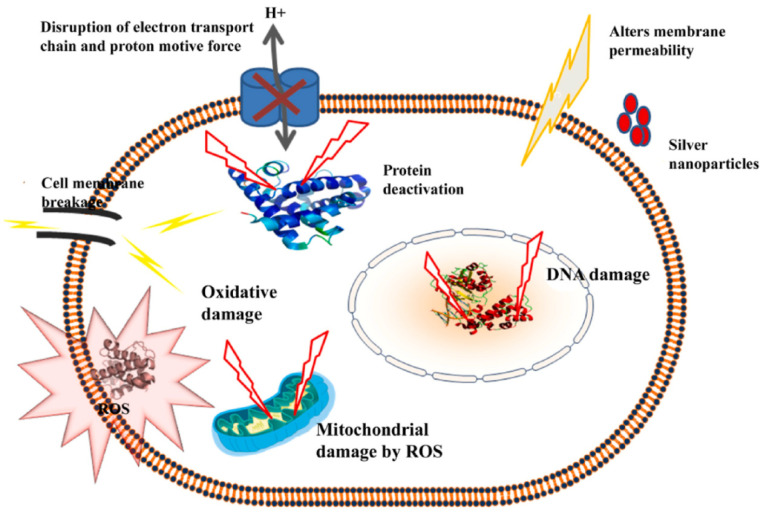
Schematic representation of proposed antimicrobial mechanism representing cell membrane leakage, disruption of electron transport chain, DNA damage and protein deactivation and Reactive oxygen *species* (ROS) production. Reproduced with permission from [63], 2023, Elsevier.

In another work, Hou et al. synthesized alginate microgel beads by insertion of silver (Ag) nanoparticles in the Na-alginate matrix. Antibacterial and apoptotic features of the product in human prostate cancer were studied [63]. Ag nanoparticles (60 nm with spherical to ellipsoidal shapes) were prepared using *perilla* plant extract, and their stability was tested by dynamic light scattering (DLS) analysis as well as zeta potential values. In this study, Na-alginate 2 wt% and 2 wt% CaCl_2_ as gelling agents were used. Through different formations including reduction/immobilization/incorporation of Ag into the matrix (in situ and ex situ methods), microgel beads were prepared (Figure 7). The thermogravimetric analysis (from 25 °C to 900 °C in flowing N_2_) was used for the examination of the manufactured nanocomposites. An initial weight loss (around 5–15%) was detected in a range of 120 °C corresponds to moisture degradation. The second decomposition showed a significant loss of mass (39–75%). The Na-alginate beads degraded around 200–300 °C and the actual weight loss was observed in the range of 320 °C and 500 °C. The antimicrobial performance of bio-nanocomposite was evaluated toward both gram-positive *B. subtilis* and *S. aureus* and gram-negative *E. coli* bacteria through the agar well diffusion technique. The antimicrobial activities were evaluated by measuring the inhibition zone. Because of its ionic nature, Na-alginate interacted with the bacterial cell wall and exerted antibacterial activity through impeding cellular functions. Ag-nanoparticles are potentially active against microbes. The antimicrobial mechanism of the composite can be seen in Figure 8. Silver ions were adsorbed via the cell membrane of bacteria and produced ROS in mitochondria resulting in oxidative stress and, lastly cell death. Additionally, in vitro cytotoxic was tested toward the five cell lines namely, HeLa, A549, LNCaP, and MCF7 carcinoma, and non-cancer IMR-90 cells were examined. Synthesized alginate/Ag nanocomposite showed promising anticancer properties. Moreover, based on the studies Na-alginate nanoparticles can penetrate Peyer’s patches on rats, making them suitable for in vivo studies owing to the great features like high encapsulation ability, green nature, biodegradability/biocompatibility, and drug delivery.

Stimuli-responsive hydrogels undergo physical or chemical changes concerning external stimuli *viz*—pH, temperature, ionic strength, light, electric, and magnetic field. Widespread investigative programs have been done on the construction of such hydrogels based on the alginate for diverse uses including water treatment. In this regard, Kumar et al. reported the successful fabricating of Na-alginate/black nano TiO_2_ bio-nanocomposite hydrogel using an ionic crosslinker [64]. Indeed, weak mechanical features, hydrophilicity, and thermal stability limit the application of hydrogel, so black TiO_2_ nanoparticles have been incorporated into the alginate matrix for the improvement in functional properties. For this aim, a Na-alginate solution (4% (*w/v*)) was first prepared at ambient temperature. TiO_2_ nanoparticles in different amounts (0, 0.02, 0.2, and 2% (*w/v*) were added to the solution and stirred to develop a uniform mixture. The homogenous solution was poured onto a glass plate. After evaporation of solvent at 60 °C, the film was obtained. Finally, the prepared film was immersed in CaCl_2_, and then it was washed and dried. The thermogravimetric analysis exhibited three steps of degradation. In the first step (below 200 °C), water molecules were removed due to the dehydration and breaking of glycosidic linkages. The second step occurred around 200–280 °C, due to alginate skeleton fracturing. With increasing temperature, carboxylate groups degraded and released CO_2_. The complete decomposition of alginate occurs at 265 °C. The decomposition temperature of the alginate improved by incorporating nano-TiO_2_ in the matrix (280 °C). After examination of bio-nanocomposite hydrogels, they were employed for the degradation of the dye. Methylene blue and malachite green were used as the model dyes (the measurements were performed at *λ*_max_ 664 nm for methylene blue and 617 nm for malachite green. Based on the outcomes, the hydrogel containing 2% (*w/v*) TiO_2_ showed the highest dye degradation potential (99% of both methylene blue (by 180 min) and malachite green (by 360 min)).

As one of the harmful pollutants in water, metal ions (Cd^2+^) require severe attention due to their accumulation in the human body through drinking water or domestic uses. In this regard, Tripathy et al. developed an effective electrochemical sensor (alginate-g-polyallylamine/reduced graphene oxide/CuO hybrid nanocomposite) for sensitive detection of Cd^2+^ in real water (River water) samples [65]. Graphene oxide was obtained via graphite oxidation in Hummer’s technique. Alginate was grafted to the polyallylamine, and a graft copolymer was organized through the free radical solution phase graft copolymerization method. This copolymer of alginate was applied for the bio-reduction of graphene oxide. –NH_2_ and –COO^−^ groups present in the macromolecular chains of the copolymer in electrostatic interaction and hydrogen bonding with the residual oxygen functionalities present in reduced graphene oxide. The obtained nanocomposite with 15.6–32.1 nm showed spindle-shaped morphology. Cyclic voltammetry and chronoamperometry analysis were employed for testing Cd^2+^ sensing. The limit of quantitation and limit of detection were 4.24 nM and 1.27 nM, respectively. Additionally, the developed sensor was stable, and reproducibility was satisfactory.

## 3. Pectin

As a natural structural fiber that is a family of complex polysaccharides, pectin is found in the primary cell wall in addition to the intracellular layer of plant cells, particularly in fruits (like oranges, apples, and lemons). This biomolecule is a family of galacturonic acid-rich polysaccharides [66,67,68]. In 1825, Henri Braconnot (a French chemist and pharmacist), discovered a heteropolysaccharide with gelling properties, which he named “pectic acid”. Among the several known biopolymers, pectin can be a good choice due to its abundance, and excellent physical and functional properties. It has many advantages, including sustainability and eco-friendliness, natural prebiotic properties, biocompatibility, biodegradability, antioxidant activity (due to carboxyl, hydroxyl, and methoxy groups), antibacterial activity, ROS scavenging activity, health benefits (anti-inflammatory and immunomodulatory), soluble dietary fiber, and gelling as well as emulsification features [69,70,71,72,73]. Therefore, pectin is a functional ingredient in the food, pharmaceutical, and cosmetic industries. Due to potential health benefits, pectins are also added as nutritional supplements. A diet rich in pectin reduces total cholesterol; additionally, it restrains the growth of the primary tumors of cancers [74,75,76,77].

**Figure 9:**
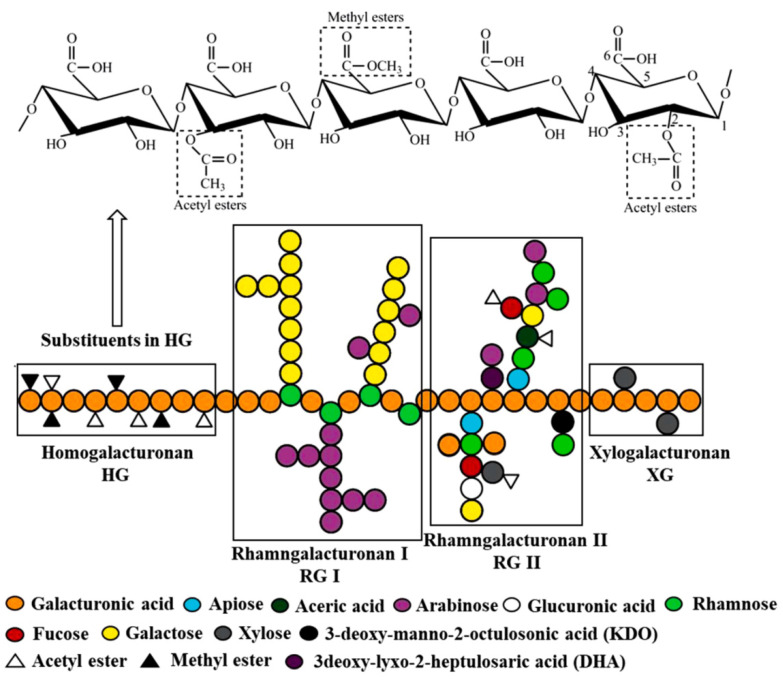
Schematic representation of pectin structure. Reproduced with permission from [77], 2020, Elsevier.

Structurally, pectin is more complex than other polysaccharides like cellulose. Pectin consists of a backbone composed of homogalacturonan (HG), rhamnogalacturonan-I (RG-I) as well as rhamnogalacuronan-II (RG-II), and in some cases, xylogalacturonan (XGA) regions (Figure 9). As “smooth region”, HG is linear homopolymer of α-1,4 linked D-galacturonic acid (GalA) units, that can be acetylated at their hydroxyl groups in position C-2 and/or C-3 and methyl-esterified at their carboxyl groups present at C-6. The ratio of methyl-esterified GalA groups to total GalA groups is quantified as a degree of esterification. Depending on the methoxyl content, pectin can be classified as high methoxyl pectin with a degree of methylation >50% (expressed as a percentage, which shows the number of methylated carboxylic functions per 100 units of galacturonic acid in the main chain) or as low methoxyl pectin with a degree of methylation <50%. The commonly used pectin refers to high methoxyl pectin. The methoxyl content reflects the dispersibility of pectin in water and its ability to form a hydrogel. Physico-chemical features and diverse applications in Low- and high-methoxyl pectins are different. The degree of methylation of the extracted pectin depends on the type of plant, its age, and degree of maturation. Therefore, pectins from fruits do not have the same degree of methylation [76,77]. Pectin has a wide range of applications like pharmaceutical, cosmetic (as an emulsifier), food (edible films, and plasticizers), biomedical, metal binding, tissue engineering, and wound healing owing to its biodegradability, biocompatibility, and non-toxicity features [69,72,73,78].

### 3.1. Source of Pectin

Pectin, as a complex anionic polysaccharide, is broadly extracted from the middle layer of plant cell walls—for example, vegetable fruit and wild fruits. Commercially, industrial pectin extraction is based on the citrus peel and apple as main sources (mostly lemon, orange, and lime because citrus peels comprise the highest quantity of it). Many other promising sources are agricultural wastes such as apple dregs, banana peel, grapefruit peel, sisal waste, pumpkin, tomato, grape, mango, watermelon rind, sunflower head, carrot, pulps (coffee and potato), and so on [66,69]. In the early 1900s, commercial construction was started. In 1926 in California, pectin was extracted from citrus fruit peel, and in 1934 in Germany, pectin was produced from dried apple pomace. Pectin achieved from different production sources shows structure diversities and chemical composition as well as different characteristics [73].

### 3.2. Methods for Pectin Extraction

**Figure 10:**
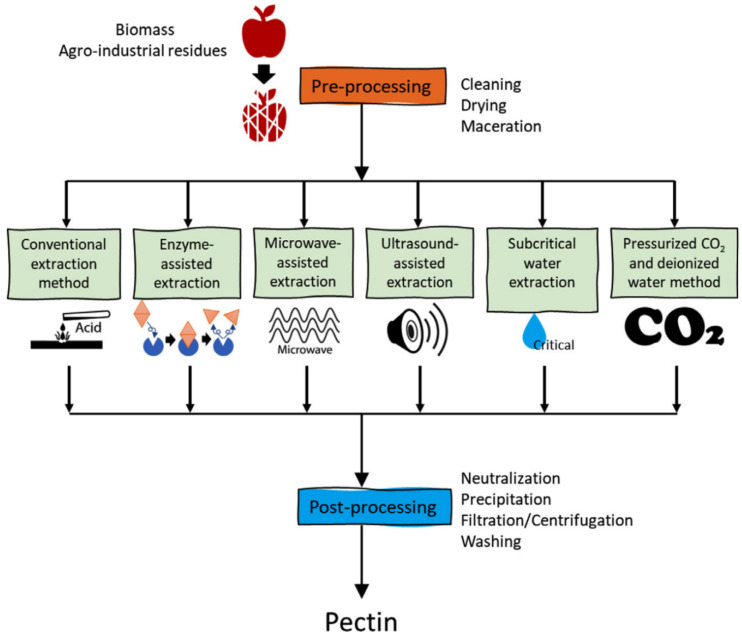
A summary of the pectin extraction process. Reproduced from [71], 2023, Elsevier, Available under CC BY license.

Pectin is extracted from various sources by a few commonly used techniques as can be seen in Figure 10. In pectin extraction methods, variables such as acid strength (if used), temperature/power, solid-to-liquid ratio, and extraction duration in the production method affect the molecular weight, composition, purity and color, and degree of esterification [71]. Pre-treatment of agro-industrial residues is usually required before the actual extraction of pectin. Although this process is optional, it affects the quality and quantity of the product. First, the residues are cleaned by washing (water-washing alcohol-washing, or acid-washing) in order to improve the extraction efficiency. After that, the drying stage is performed. In addition, grinding the material into powder increases the surface area and better extraction [66,67].

Conventional (or acid-mediated) extraction: This treatment method involves low pH and high temperature (1.5–3.0 pH, 75–100 °C) of pectin source for up to 3 h. In fact, hydrolysis of protopectin into pectin and solubilization of the pectin are achieved by heating the raw materials in acidified buffers. Strong and highly corrosive mineral acids, like HNO_3_, HCl, and H_2_SO_4_, have been broadly used. However, they have many drawbacks, such as extraction costs and environmental issues. Mild food-grade organic acids, including acetic, citric, oxalic, and tartaric acids, addressed these problems. Additionally, citric acid as a chelating agent with great performance showed the highest pectin production yield (14.34%) from potato pulp [67,68,71]. In the enzyme-assisted extraction method, enzymes are used to break down and degrade the cell wall matrix for pectin release. The most general enzymes are protopectinases, cellulases, proteases, *α*-amylase, *β*-glucanase, hemicellulases, and xylanases. This method has many advantages, including eco-friendliness, high selectivity, high yield, low temperature and low energy consumption, and avoiding damaging chemicals such as acids. However, this technique is more costly and time-consuming, and enzymes are sensitive to chemical compositions, temperature, and pH [71]. The microwave-based method involves heat generation when the dielectric materials are exposed to microwave radiation. The extraction period is short, and it is more time-saving than the conventional process. However, an increase in microwave power or irradiation duration might lead to degraded pectin and lower yield [71]. In the ultrasound-assisted extraction process, sound waves in the range of 20–40 kHz are used for pectin extraction. The irradiation of the raw materials with ultrasound served multiple purposes. In fact, pressure fluctuations produced by ultrasonic vibrations form microbubbles (microjet) for the disruption of cell walls. Many advantages of this process are lower extraction time, high yield, and environmental friendliness compared to traditional extraction methods [67].

In recent years, subcritical water has been progressively employed for pectin extraction. Subcritical water (or “pressurized hot water”: liquid water under pressure at temperatures above the usual boiling point, 100 °C. In a subcritical state, water is maintained in liquid form by applying pressure) can assist the pectin extraction from the agro-industrial residues. Subcritical water is water with a liquid state and a temperature between 100 °C and 374 °C (critical point). This method is eco-friendly, economical, and safer than conventional acid extraction. However, pectin degradation may occur when the temperature is higher than a certain amount (depending on the pectin composition found in the raw materials, and heat-sensitive components) [66]. On the other side, as a novel extraction technique, pressurized carbon dioxide and deionized water methods can acidify water and also generate carbonate ions (a natural chelate of calcium ions). The method is underdeveloped, however, it has a lot of potential because of its low-emission nature [66]. Additionally, the aim of post-extraction processing is based on removing or neutralizing the chemicals mixed with the pectin in addition to removing/replacing the buffer/solvent before further applications. After filtering the extract solid solubilized pectin should be precipitated by mixing it with alcohol (60% ethanol) [71].

### 3.3. Pectin-Based Bio-Nanocomposites for Diverse Applications

**Figure 11:**
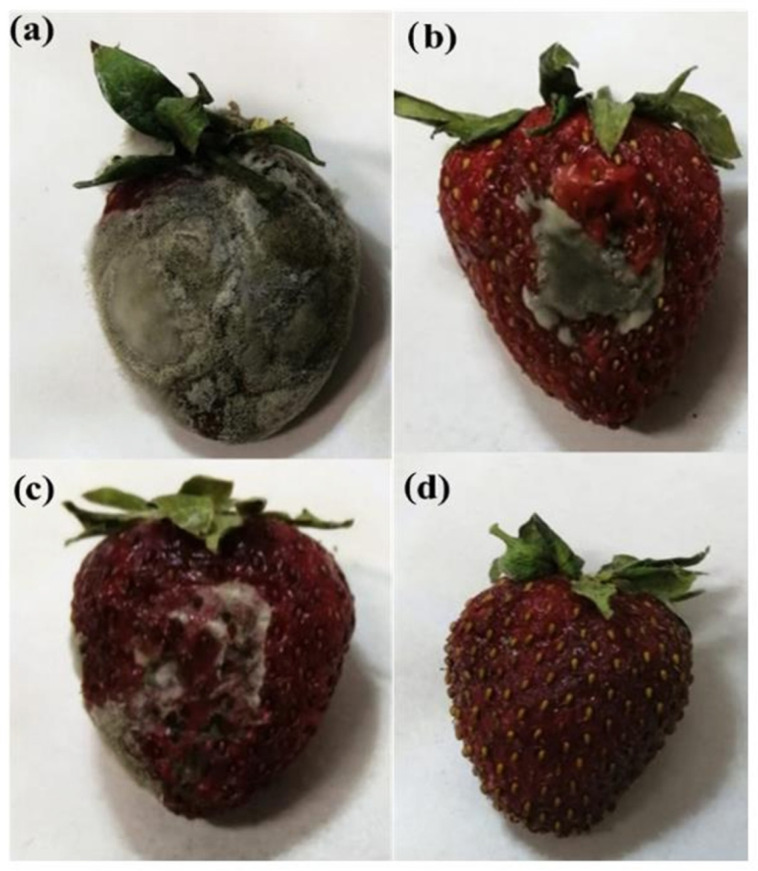
Fresh strawberry: (**a**) uncoated; (**b**) coated with P-H80G20; (**c**) coated with P-H70G30, and (**d**) coated with P-H50G50 after 10 days of storage at room temperature, RH = 60% (pectin from apple (P), halloysite nanotubes (H), grapefruit seed oil (G), nano-hybrids HxGy). Reproduced from [79], 2022, MDPI, available under CC BY license.

For the production of active and edible packaging, in addition to being economical, the migration of gases, moisture, and lipids should be controlled. Among polysaccharides, pectin has been verified to be an outstanding material for use as a coating for food protection due to its film-forming ability. However, many physical characteristics like barrier and antimicrobial performance of pectin films are poor, so filling pectin with antimicrobials like essential oils is a fascinating technique. In this regard, Viscusi et al. fabricated and characterized the bio-nanocomposites using halloysite nanotubes-encapsulating grapefruit seed oil in a pectin matrix. This product was used as bio-coating for the preservation of fresh fruits (strawberries) [79]. Grapefruit seed oil has antifungal, antioxidant, and anticancer features and shows antimicrobial action against foodborne pathogens. For the preparation of bio-nanocomposite, first, grapefruit seed oil (in different amounts of 20, 30, and 50% *w*/*w*) was added to the halloysite nanotubes, and this nano-hybrid was placed in the vacuum environment to remove air. After dissolving in water and adding a plasticizer (glycerol), a pectin–glycerol solution was obtained. After adding nano-hybrid to this solution, the mixture was poured into Petri dishes and cast at room temperature. Mechanical, optical, barrier, and thermal features of the active coating were examined. Based on thermogravimetric analysis, pectin and pectin composites displayed a three-step degradation behavior. The first step up to ~150 °C was associated with water evaporation. The second step (around 250 °C) was related to the pyrolytic decomposition. The last stage was related to the oxidation process. Mechanical presentations were estimated by carrying out stress-strain assessments. Compared to the pure pectin film, the prepared films showed an increase in water permeability up to 480%, a 200% increase in elastic modulus, stress at breaking 48%, and deformation at breaking up to 39%. An enhancement of the elastic modulus of pectin composites was observed in compliance with the amount of halloysite nanotubes (26%, 49%, and 67%). This phenomenon was related to the reinforcement effect of halloysite nanotubes through the -H bond formation. After loading the hybrid filler, the strength at the break point increased. These consequences showed the good dispersion of hybrid filler and the absence of cluster formation. To study the efficiency of bio-nanocomposite as an active coating, fresh strawberries were covered by them. As can be observed in Figure 11, after two days, uncoated strawberries were completely covered by mold and damaged, while nanocomposite with 50% *w*/*w* grapefruit seed oil showed full protection of the strawberry from degradation.

Polymer blending is an efficient way to prepare desired polymeric materials in a wide range of applications. Blending pectin with other polymers like polyvinyl alcohol or adding hydrophobic materials like essential oils and reinforcing with nanofillers are good ways to advance the barrier properties of pectin. Suhasini et al. prepared pectin-based films for food packaging uses. In this study, nanocomposite films of pectin/polyvinyl alcohol and pectin-MgO/polyvinyl alcohol were prepared by casting technique [80]. MgO nanoparticles were inserted into the matrix to develop thermal stability and mechanical performance. Additionally, the insertion of MgO nanoparticles into the pectin matrix improves the antimicrobial performance. For this aim, the extraction of pectin from citrus fruit peel was achieved using the solvent precipitation technique. The freshly gathered peels of each fruit were washed multiple times with tap water to remove dirt and debris from the surface of citrus fruits, and peel powders were mixed with distilled water. pH value was adjusted at 1.06, by adding HCl. This mixture was heated (80 °C), and then, it was cooled and filtered. The collected filtrate was mixed with ethanol to prepare pectin (12% wt yield, 166.7 g/mol, 66.6% esterification, 9.92% methoxy content). Pectin/polyvinyl alcohol and pectin-MgO/polyvinyl alcohol films were organized through the casting method. For the preparation of the film, about 0.5 g of pectin powder and 0.15 g of polyvinyl alcohol was used. Additionally, 2.4 g of Mg(NO_3_)_2_⋅6H_2_O was used for the nanocomposite. The resulting films displayed good thermal stability (200 °C). Bio-nanocomposite film with MgO displayed increased antioxidant activity, low solubility, and decreased water vapor permeability. Using a screw gauge, the thicknesses of nanocomposite films were measured. Pectin/polyvinyl alcohol and pectin-MgO/polyvinyl alcohol films showed 23 μm and 29 μm thicknesses, respectively. Additionally, the bio-nanocomposite film exhibited developed tensile strength and modulus compared to pectin/polyvinyl alcohol due to the existence of MgO nanoparticles, which reinforced the films, while pectin/polyvinyl alcohol showed higher elongation and more flexibility than the bio-nanocomposite film. Based on the TGA-DTA thermogram, four stages of weight loss were observed. The first step (weight loss of 15.96%), in the 32.19–124.76 °C range, was owing to the elimination of water/volatiles. The second step (43.39% weight loss), from 124.76–230.71 °C, was due to depolymerization of the polymer chains. The percentage of weight loss in this step for bio-nanocomposite was less compared to the polymeric matrix because of the intense interaction of MgO with the matrix. The next step (26.79% loss), from 230.7–542.2 °C, was due to the breakdown of polyvinyl alcohol. The last residue (9.25%) at 900 °C was attributed to the MgO. Due to the presence of MgO nanoparticles, the thermal properties of the fabricated nanocomposite were better than pectin/polyvinyl alcohol. Bio-degradation studies in soil and water were conducted. In soil, they exhibited no change in the weight of the sample for the initial 24 h, but after one week, a 21.5% weight reduction for pectin/polyvinyl alcohol film and a 13.6% reduction for pectin-MgO/polyvinyl alcohol were observed due to soil-resident bacteria. In plain water and seawater, on the 12th day, the pectin/polyvinyl alcohol film started to fragment and its nanocomposite started to decolorize. On the 30th day, the pectin/polyvinyl alcohol film started to dissolve. Nevertheless, in nanocomposite film, no such changes were seen. As a point, in the rate of degradation of polymers in water, several features including the presence of microorganisms in water, agitation of media, the water-to-film weight ratio, besides oxygenation of media are important. After that time, it was found that both films were quite fragmented, presenting a flaky appearance and completely lost their initial shape. In another study by Souza et al. [81] many pectin-based films were prepared by the insertion of cellulose nanocrystals, sodium montmorillonite nanoparticles, and an additional chitosan layer for food packaging. Well-dispersed and exfoliated montmorillonite (with stacked silicate sheets) is used to improve the mechanical characteristics of different bio-films. Additionally, organic fillers like cellulose nanocrystals are used in mechanical improvements. To progress the barrier and mechanical characteristics of bio-films, a combination of two or more bio-polymers in blends or layers is used; 2.5% wt. of montmorillonite and cellulose nanocrystals were used in the pectin solution. For this aim, pectin was dissolved in water containing glycerol. Ultrasonication was applied to prepare a homogenized solution. After that, fillers were added individually to the solution of pectin and agitated in an ultrasound batch. After the casting of solutions on glass plates and drying, the bio-composite films were produced. The insertion of fillers reduced water vapor permeability and improved their contact angle. Cellulose nanocrystals increased modulus of elasticity and tensile strength. The mechanical barrier, as well as the optical features, were examined and compared to pure film. All pectin-based films showed higher oxygen barrier features than polyethylene, which is extensively used in food packaging. In this work, the chitosan layer did not enhance the mechanical features of the pectin but made the material more hydrophobic, which played a significant role in packaging.

Przybyszewska et al. produced pectin-based bio-nanocomposite film via the incorporation of ZnO nanoparticles with respectable microbiological and antioxidant features against the growth of *Enterobacteriaceae* during refrigerated storing of fresh chick meat [82]. For this aim, at first, ZnO nanoparticles were fabricated in a green method by extracts of tomato and passion fruit. For this aim, zinc nitrate was added to the extract solutions. Then, KOH solution was added for the ZnO precipitate. A uniform-film-forming solution containing apple pectin, ZnO nanoparticles (5% *w*/*w* relative to pectin), and plasticizer (glycerol, 30% relative to pectin) were prepared. This solution was heated (20 min at 60 °C and 250 rpm magnetic stirrer). Then, it was poured as films on sheets using an automatic film applicator and dried. For testing the films, fresh meat was obtained from a supermarket. It was placed in the produced film and placed in a plastic container in the refrigerator for 15 days. A control sample (un-packed meat) also was served and examined. Based on the outcomes, the bio-nanocomposites presented good antimicrobial performance. Antioxidant activity (evaluated by the Thiobarbituric Acid Reactive Substances (TBARS) values) presented an extension of shelf life time.

Candido and co-workers designed pectin bio-nanocomposites by insertion of pracaxi oil nano-emulsion and served as active packaging with antioxidant action for butter [83]. Nano-emulsions of pracaxi oil were produced through ultrasonication. Water vapor permeation and thermodynamic parameters in the films were examined. A solution of highly methoxylated citrus pectin (3%) was prepared in pracaxi oil oil-in-water emulsions and homogenized via a magnetic stirrer. After the addition of xylitol and stirring for another 1 h, they were poured into a Petri plate and dried. The antioxidant oil with the presence of phenolic compounds efficiently enhanced the butter stability against oxidation processes during the 60 days of storage. The antioxidant performance was through DPPH, phosphomolybdenum, and β-carotene protection analysis.

**Figure 12:**
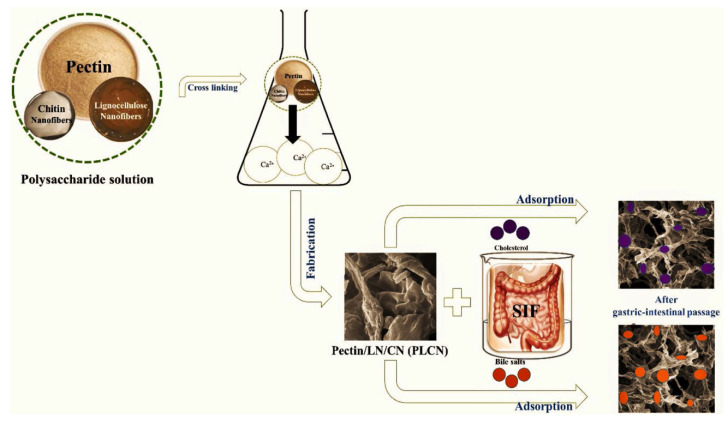
Schematic illustration for the fabrication of pectin/lignocellulose nanofibers/chitin nanofibers and its application in cholesterol and bile salts adsorption from simulated intestinal fluid. Reproduced with permission from [84], 2021, Elsevier.

Khorasani and their research team reported the preparation of pectin/lignocellulose/chitin bio-nanocomposite (52%, 31%, and 17%, respectively in 1% (*w/v*) homogenous polysaccharide mixture) as an effective bio-sorbent for adsorption of cholesterol and bile salts from simulated intestinal fluid during gastric-intestinal passageway for medicine and food uses. A mixture of pectin, lignocellulose, and chitin could be a multifunctional choice since it is a combination of gastrointestinal-resistant material for probiotic delivery and a good candidate for the removal of cholesterol and bile salts [84]. For this aim, suspensions of pectin, lignocellulose nanofibers, and chitin nanofibers were prepared by adding them to water. They were mixed and agitated (at 60 °C) for preparation of a uniform polysaccharide mixture. By adding the mixture dropwise into a crosslinking agent (CaCl_2_ solution 5%) and keeping it at 4 °C for 60 min, a hard matrix was produced. Finally, it was centrifuged and freeze-dried. A bio-nanocomposite with irregular shape morphology and a wrinkled surface was prepared by van der Waals and hydrogen bonding (between nanofibers and pectin) as well as ionic crosslinking (between Ca^2+^ and pectin (–COO^−^) groups). Nanofibers with specific surface areas improved the bio-composite stability and adsorption performance. In vitro evaluation of bio-nanocomposite for adsorption can be seen in Figure 12. The maximum adsorption capacity was 5578.4 mg/g bile salts and 37.9 mg/g cholesterol. The Freundlich isotherm showed the reversible heterogeneous adsorption, and it proved the physical and chemical adsorption mechanism for cholesterol and bile salts, respectively.

**Figure 13:**
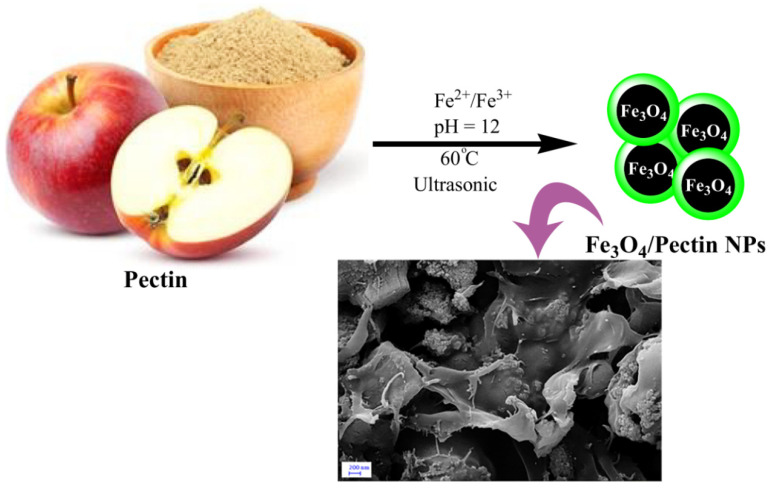
Schematic one-pot preparation of Fe_3_O_4_/pectin nanoparticles under ultrasound conditions, and FE-SEM images of Fe_3_O_4_/Pectin. The scale bar represents a distance of 200 nm. Adapted from [85], 2022, Elsevier, available under CC BY license.

Wang et al. reported the one-pot production of apple pectin encapsulated with Fe_3_O_4_ nanoparticles used to cure colorectal cancer [85]. For this aim, a solution of pectin was prepared by dissolving 0.2 g of apple pectin in 50 mL DI water. Fe_3_O_4_/Pectin prepared by co-precipitation of Fe ions in a basic solution mediated by pectin in an ultrasound situation (Figure 13). An amount of 0.34 g of Fe ions was added into the pectin solution. Under ultrasonic conditions, NaOH was added (pH: 12). It was magnetized, and finally, Fe_3_O_4_/pectin were collected and washed. The product as an antihuman colorectal carcinoma, the bio-nanocomposite exhibited high antioxidant activity toward DPPH (a free radical that changes color in the presence of substances with antioxidant properties and captures electrons).

**Figure 14:**
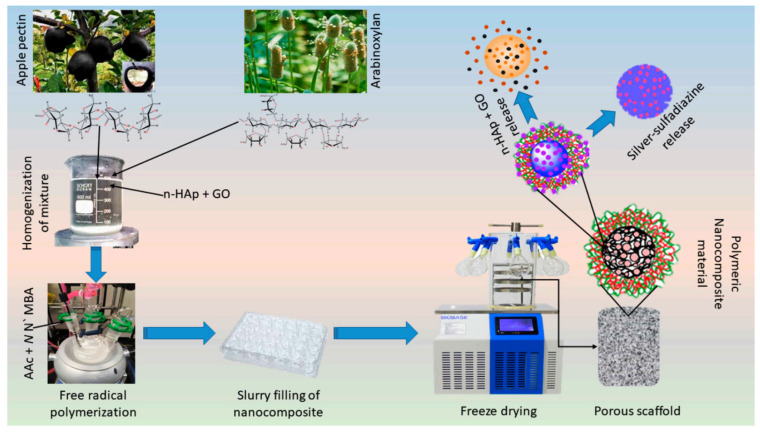
Experimental design. Polymeric nanocomposite scaffolds were synthesized by free radical polymerization to fabricate porous scaffolds via the freeze-drying method. Briefly, biopolymers (arabinoxylan, apple pectin), acrylic acid (monomer), n-Hydroxyapatite, and graphene oxide were stirred to have a homogenized mixture and crosslinked by using *N*,*N*0-methylene-bis-acrylamide to form the hybrid nanocomposite. These hybrid nanocomposites were then freeze-dried to have porous scaffolds. Finally, the mouse pre-osteoblast (*MC3T3-E1*) cell line was used to evaluate in vitro behavior of these scaffolds. Reproduced from [86], 2020, MDPI, available under CC BY license.

In an investigation by Al-Arjan et al., a porous foam-like nanocomposite scaffold was produced by a free-radical polymerization process using apple pectin, arabinoxylan, hydroxyapatite, and graphene oxide. For this aim, 1.0 g apple pectin and 1.0 g arabinoxylan were dispersed and added into a three-necked round-bottom flask. Acrylic acid monomer and acrylamide crosslinker were added to the solution; 2 g nano-hydroxyapatite and 0.1, 0.2, 0.3, and 0.4 mg of graphene oxide were slowly added and stirred. After adding the K_2_S_2_O_8_ initiator and stirring, the reaction was stopped by removing the flow of nitrogen gas [86]. Graphene oxide improved physicochemical as well as biomechanical features (Figure 14). Using mouse pre-osteoblast (MC3T3-E1) cell lines, the in vitro analysis was performed using mouse pre-osteoblast (MC3T3-E1) cell lines. This bio-nanocomposite showed interesting bone scaffold regeneration features and excellent biocompatibility. This part is summarized in Table 3.

## 4. Chitin and Chitosan

**Figure 15:**
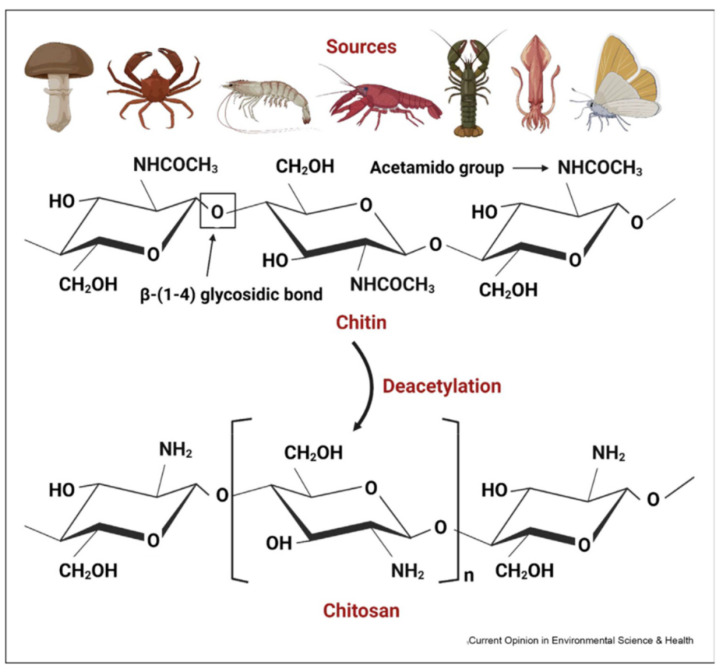
Structure of chitin and chitosan. Reproduced with permission from [94], 2023, Elsevier.

**Figure 16:**
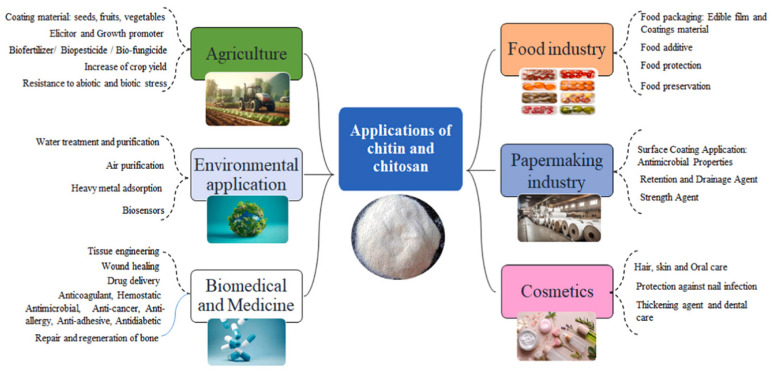
Exploring diverse applications of chitin and chitosan. Reproduced with permission from [103], 2024, Elsevier.

As a linear amino-polysaccharide and second most abundant natural biopolymer (after cellulose) with unique chemical, physical, and biological features, chitin (C_8_H_13_O_5_N) is made up of *β*-D-N-acetylglucosamine units linked with *β* (1→4) bonds. Because of the existence of β bonds positioned between C-1 and C-4, it is a rigid polysaccharide. It is a non-toxic, biocompatible, and biodegradable polymer that has three different (including α, β, and γ.) crystalline allomorphs [87,88,89]. The α-chitin (most abundant, stable, easily accessible, strong intermolecular H bonds), β-chitin (weaker intermolecular forces, lower stability), and γ-chitin are made of antiparallel, parallel, as well as parallel/antiparallel arrangements, respectively. Overall, owing to inter- and intramolecular hydrogen bonding systems, chitin is insoluble in inorganic and organic solvents [87]. On the other side, chitosan (C_6_H_11_O_4_N), is a polycationic co-polymer containing β-(1→4)-2-acetamido-D-glucbose and β-(1→4)-2-amino-D-glucose parts (two usual sugars), and is the deacetylation product of chitin (Figure 15) [90,91,92,93,94,95]. The deacetylation degree is the most important characteristic of chitosan. Deacetylation degrees of 55–70%, 70–85%, and 85–95% are defined as a low deacetylated degree, middle deacetylation degree, and high deacetylation degree of chitosan, respectively, which are almost completely insoluble, partly dissolved, and have good solubility in water. Values of 95–100% are called the ultrahigh deacetylation degree of chitosan, which is difficult to achieve. The structural difference in chitin and chitosan fabrications is in the side groups at the C2 position (chitin contains an amino group and chitosan has an *N*-acetyl amine group in the backbone) [96]. Based on various sources, chitosan has different MW values: low MW (50–90 kDa), medium (190–310 kDa), and high MW (310–375 kDa). Chitosan with low MW is generally favorable for the pharmaceutical areas [87]. Chitosan has many great characteristics like biodegradability, chemically active (amine/hydroxyl groups) due to abundant resources, non-toxicity, low cost, biocompatibility, antimicrobial antioxidant properties, and appropriate biological properties. It is soluble in aqueous acid solutions (acidic aqueous solutions below pH 6.3), but insoluble in water and organic solvents. Chitosan and its derivatives have been employed in various fields including biomedical, wound healing, food, cosmetics, tissue engineering, agriculture, wastewater treatment, sensors, and so on (Figure 16) [97,98,99,100,101,102,103].

### 4.1. Source and Extraction Techniques of Chitin and Chitosan

Chitin is present in a vast variety of sources. It is mostly abundant in the crustacean’s shells and marine exoskeleton (like shrimp, crabs, arthropods, prawns, crustaceans, krill, cephalopods, shrimps, mollusks, and lobsters), arthropod cuticles, and cell walls of fungi, yeasts, and insects. For bulk production of chitin and chitosan, marine sources as raw materials are used [104,105]. However, in recent years, alternative sources like microbial biomass, insect biomass, mushroom/fungal, and cell walls have received increased interest. The source of chitin, reaction conditions, and deacetylation degree are the key factors for the determination of MW of chitosan and its functional features. Chitin and chitosan are extracted by chemical and biological approaches and sometimes, a combination of the methods may be performed that is summarized in Table 4 [87,88].

**Figure 17:**
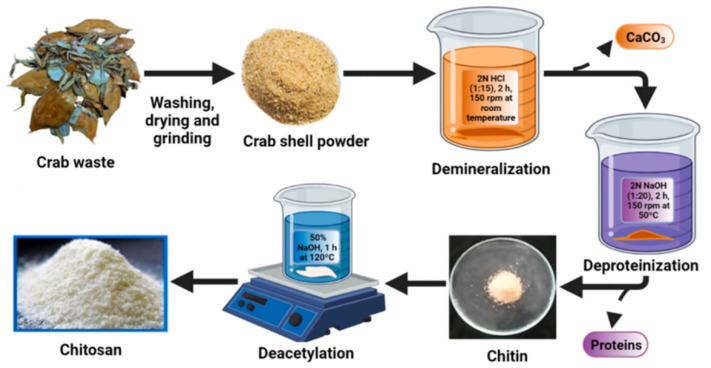
Schematic diagram of chemical extraction. Reproduced with permission from [87], 2022, Elsevier.

In the conventional (chemical extraction) method, acidic and alkaline solvents (NaOH > 40% wt) and high temperatures (>100 °C) over a long time period are used to extract chitin and chitosan from crustacean shell waste, mollusks, insects, and fungi. The chemical procedure is the most common and is very applied for commercial purposes. Preparation of chitosan by this method includes three reaction steps: (1) demineralization (minerals mostly CaCO_3_ in the shell are removed through the treatment of strong acids (H_2_SO_4_, HNO_3_, HCl, HCOOH) for 2–3 h with agitation, (2) deproteination—using chemicals (NaOH, KOH as deproteinizing agents) to break the chemical connection among the protein and the chitin (removal of protein and other organic components other than chitin) at temperatures of 65–100 °C; and (3) deacetylation—amides undergo hydrolysis in acidic or alkaline solutions (Figure 17) [87,88]. For example, Hisham et al. extracted chitin and chitosan from shrimp shells via two-step acid and alkaline treatments. NaOH was used to deproteinize shrimp shells, and 1%, 2%, 3%, 4%, and 5% of hydrochloric acid was used to demineralize. Based on the FT-IR results, α-chitin isomorph was extracted from shrimp shells. With an increase in acid concentration, the surface morphology of chitin increased. Chitosan with 65% deacetylation degree and with rod-like micro-structure was effectively produced using this chemical way. The produced chitosan could dissolve in the 2% (*w/v*) acetic acid [106].

**Figure 18:**
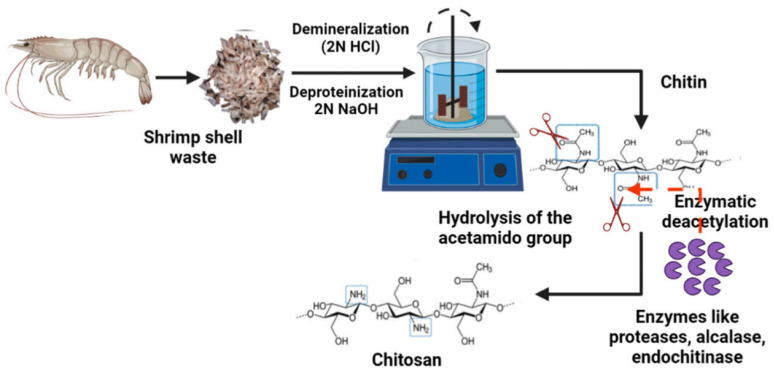
Schematic diagram of enzyme-assisted extraction. Reproduced with permission from [87], 2022, Elsevier.

Apart from the chemical approach, green methods including microbial fermentation, microwave technology, and enzymatic methods exist for the preparation of chitosan. Indeed, to overcome the problem of chemical methods (which affect the physicochemical features of chitosan), bio-based approaches have been developed. In enzyme-assisted extraction, a demineralization mechanism is performed using acid to remove the CaCO_3_ in a shell. The deproteinization and deacetylation reactions are performed with enzymes at milder temperatures (25–59 °C) (Figure 18) [87]. Enzymes are commonly extracted from microbes or fish entrails. The use of enzymes avoids irregular deacetylation as well as MW reduction (as the red arrow shows in this Figure 18, in the presence of enzymes, which is marked with purple color, chitin deacetylation is carried out). Chitinases, proteases, and carbohydrases are examples of such enzymes. Despite the benefit of extraction under mild conditions, this method still has many limits for industrial-scale production, concerning the high price of enzymes used for deproteinization and deacetylation in contrast to affordable chemical reagents [87,88].

For example, Hongkulsup and co-workers [107] reported enzyme-assisted extraction of chitin from the shrimp shells ((*Litopenaeus vannamei*)). The effect of process conditions, including enzyme–substrate ratio and incubation time, was examined on process efficiency and yields. Deproteinized shells were treated with 0.5 M lactic acid and 0.5 M acetic acid. in the case of lactic and acetic acids, demineralization improved (time: 20 min) to maximum values of 73.85% and 65.66%, respectively. The greater percentage of demineralization by lactic acid was due to being a stronger acid (pKa = 3.86) than acetic acid (pKa = 4.76). Based on the results, demineralization values were 98.64 and 97.57% for lactic and acetic acids at 25 °C, 20 min time, shells–lactic acid ratio of 1:1.1 *w*/*w*; and shells–acetic acid ratio of 1:1.2 *w*/*w*.

To resolve the high cost of the enzymes, an alternative method (microbial fermentation) has been advanced. This method has two sub-categories: lactic acid (microbial strains used in the studies secrete lactic acid for demineralization) and non-lactic acid (other organic acids) fermentation methods. Generally, although chemical methods have many benefits including shorter process times and simpler manufacturing procedures, the produced chitosan has lower MW and a higher degree of deacetylation, uses toxic or corrosive chemicals like HCl, and producing by-products are disadvantages of them. While biological methods produce chitosan with high MW and better mechanical performance [87]. Nian Tan et al. reported microbial extraction of chitin from seafood waste using sugars derived from fruit waste-stream [108]. *Lactobacillus plantarum* subsp. *plantarum* ATCC 14917 and *Bacillus subtilis* subsp. *Subtilis* ATCC 6051 were employed for the co-fermentation of shrimp waste. After deproteinization/demineralization of the prawn shells, chitin was effectively extracted. For fermentation, waste substrate glucose supplementation was used and reduced cost and maximized waste uses. Sugarcane molasses, light corn syrup, red grape pomace, white grape pomace, apple peel, pineapple peel and core, potato peel, mango peel, banana peel, and sweet potato peel as carbon sources were examined and compared. Chitin extracted from fermentation with potato peel had the highest dry weight of 0.89 g. Recovery of chitin with red grape pomace resulted in a 6.85 C/N ratio, 72.90% deacetylation degree, and 95.54% crystallinity degree.

Microwave-assisted extraction method has several advantages, including low process cost, short heating, energy efficiency, easy accurate process control, and selective heating. Microwave heating has been employed for polysaccharides extraction like chitosan and reduced the usage of the chemicals. In this method, several conditions like solvent concentration, solid-to-liquid ratio, and reaction time have an effect on MW and degree of deacetylation, so optimizing the conditions is critical. The produced chitosan from microwave heating showed higher MW compared to the conventional heating method, but the same crystallinity, structures, and morphologies [87].

### 4.2. Chitosan-Based Bio-Nanocomposites

**Figure 19:**
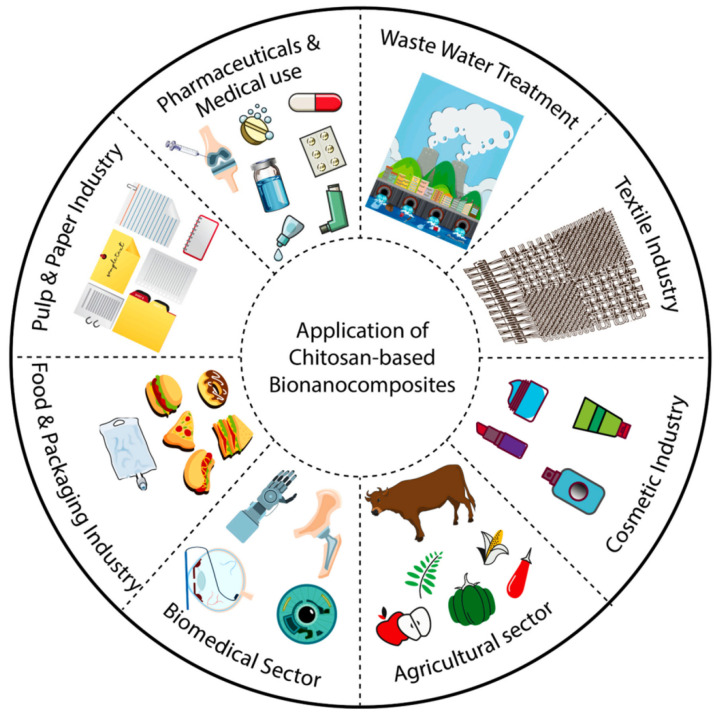
Broadened application of chitosan-based bio-nanocomposites. Reproduced with permission from [105], 2021, Elsevier.

Owing to its polycationic nature, biocompatibility, non-toxicity, versatility, as well as great physicochemical features, chitosan has assembled consideration from researchers over the years. By incorporating nano-sized fillers into the chitosan matrix, bio-nanocomposites have been identified [105]. Chitosan-based bio-nanocomposites have been used in numerous industrial areas like food processing (anti-microbial packaging or emulsifier), cosmetic, textile, biosensor, bioimaging, cancer therapy, wastewater treatment, tissue engineering, advanced wound care, pharmaceuticals, and agriculture industries (Figure 19) [105]. The most noticeable characteristic of chitosan-based bio-nanocomposites is bio-compatibility. This feature exerts concerning the -NH_3_^+^ and the negatively charged groups via H bonds and electrostatic attraction. Another feature of bio-nanocomposites is low/non-toxicity. Chitosan nanocomposites decompose in aerobic or anaerobic conditions because of the microorganism (or enzyme) actions. Capable of forming thin films, thermal stability, excellent mechanical and barrier facility, and strong antimicrobial/antioxidant/antifungal potential are other advantages of bio-nanocomposites based on chitosan. However, low solubility (limitation for the medicinal field), low colloidal stability (for drug delivery on a larger scale), high elasticity, and economic problems for the real market are many drawbacks. Typical approaches normally applied to make these bio-nanocomposites include the solution-casting method as the easiest process to create polymeric nanocomposite), in situ technique (an effective technique for producing composites with homogenously distributed nanofiller), electrospinning technique (can generate thin composite fibers), and freeze drying technique [97,105]. In the following, the introduction of the latest (in 2023 and 2024) bio-nanocomposites based on chitosan for various applications is mentioned and summarized in Table 5.

**Figure 20:**
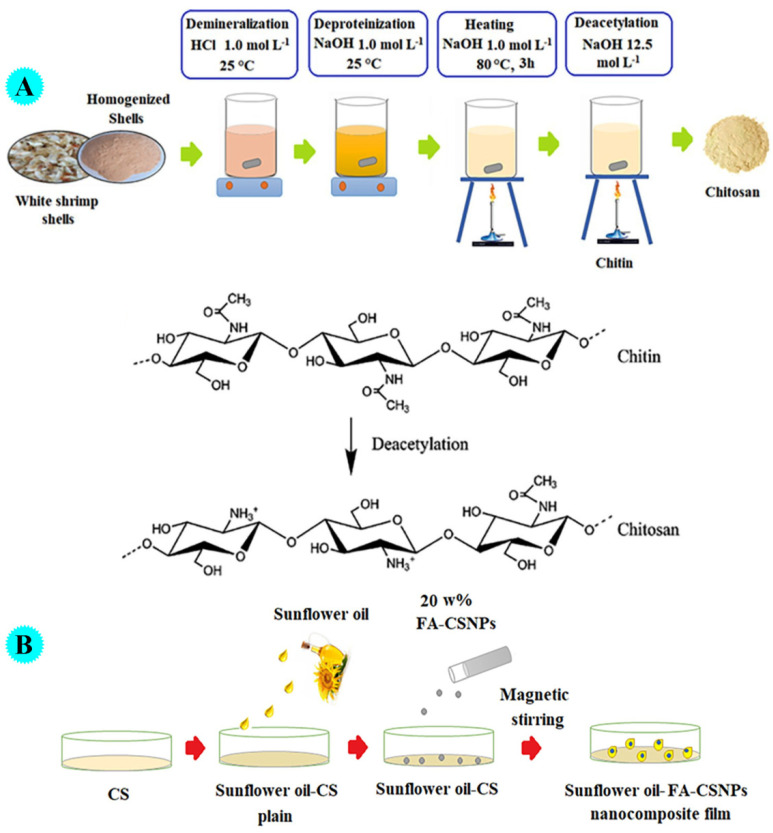
Isolation of chitin and formation of chitosan (**A**), Synthesis of sunflower oil/chitosan/fly ash bio-nanocomposite film (**B**). Reproduced from [109], 2023, PLOS ONE, available under CC BY license.

Alterary et al. produced bio-nanocomposite film based on sunflower oil, chitosan, and fly ash, and examined its antibacterial as well as immunomodulatory potential [109]. For this aim, at first, chitosan was extracted from white shrimp (*Litopenaeus vannamei*). By the addition of HCl at room temperature, shrimp shells were demineralized (calcium carbonate and phosphate were removed). After filtering and washing to reach neutralized pH, they were immersed in 95% ethanol and a pure crystalline form was obtained and dried. By adding NaOH, demineralized shells were deproteinized. The mixture was heated, filtered, and washed with water. After further bleaching, it was immersed in ethanol, and chitin was obtained. After deacetylation by treating with NaOH, freezing, and heating at 120 °C, chitosan was formed, filtered, neutralized, and dried. In the next step, chitosan was dissolved in CH_3_COOH (glacial) and ash extract solution was added. By adding (drop by drop) the mixture into a crosslinker solution (tripolyphosphate), chitosan/fly ash nanoparticles were attained. For the preparation of sunflower oil/chitosan/fly ash bio-nanocomposite, a solution of chitosan was prepared, and sunflower oil (2% *w/v*) was added (at room temperature) and stirred. Finally, chitosan/fly ash was added under agitation, and uniform (without cracks and pores) sunflower oil/chitosan/fly ash film was achieved (Figure 20). The construction of bio-nanocomposite film was confirmed via diverse spectroscopic and microscopic analyses. Thermogravimetric analysis (between 30 °C to 500 °C under the N_2_ gas atmosphere) was used for the measurement of the thermal stability of the bio-nanocomposite film. The main decomposition step began at 295 °C and 310 °C with a maximum decomposition rate at 345 °C and 370 °C, mainly due to the degradation of the long biopolymeric chain. The outcomes proposed that the sunflower oil/chitosan/fly ash film bio-nanocomposite film displayed stability up to 380 °C, which was higher than the individual components because of the strong intermolecular interactions between them. The designed bio-nanocomposite revealed favorable antibacterial action to *Pseudomonas aeruginosa* (28 mm inhibition zone) and *Bacillus subtilis* (34 mm inhibition zone) and a high (98.95%) cell viability effect. Antibacterial activity by the agar diffusion method could be achieved using two possible mechanisms: generation of ROS or electrostatic interaction among the nanocomposite and cell membrane. Additionally, a direct attack on the membrane and damage of protein, DNA, and lipids causes death. The immunomodulatory influence of the pre-fabricated nanocomposite was examined on RAW264.7 cells and the outcomes showed outstanding immunomodulatory potential through promoting phagocytosis and enhancing the construction of cytokines.

**Figure 21:**
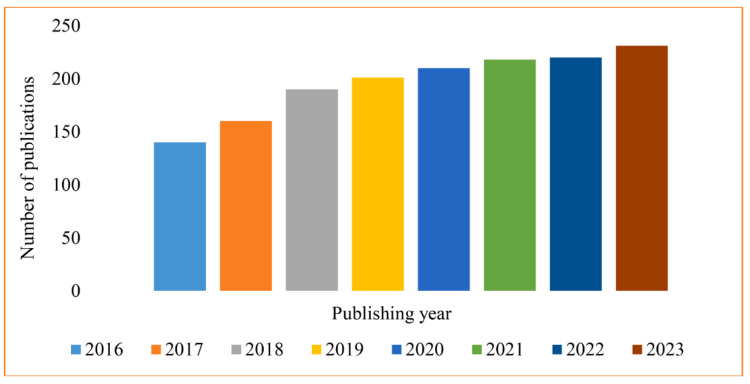
A number of published papers in recent years focused on the chitosan-based bio-nanocomposites for wastewater treatment (the data has been collected from Scopus database). Reproduced from [110], 2024, Elsevier, available under CC BY license.

Chitosan-based nanocomposite has attracted widespread attention for the adsorption of pollutants from water using enlarging the peripheral surface area, porosity, pore volume, and holding/possessing of the more active sites or functional groups for example –NH_2_, –OH, and –COOH. The rapidly increasing tendency of research publications to focus on chitosan-based nanocomposite filters lately (Scopus database) can be seen in Figure 21 [110].

**Figure 22:**
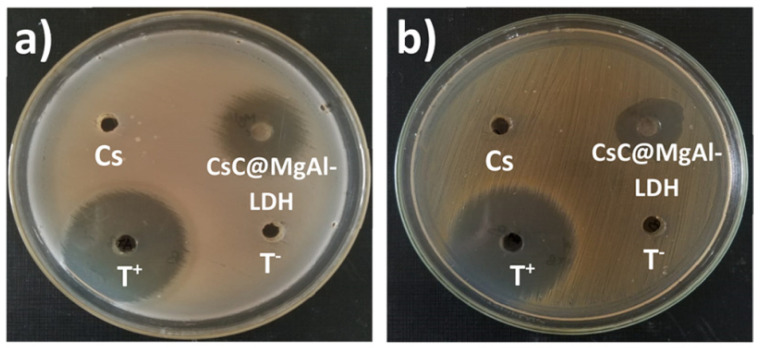

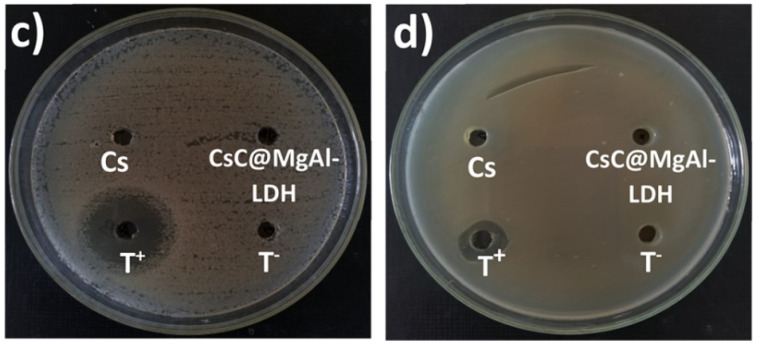
Antibacterial activity of CsC@MgAl-LDH against *Bacillus subtilis* (**a**), *Staphylococcus aureus* (**b**), *Escherichia coli* (**c**), and *Pseudomonas aeruginosa* (**d**). Reproduced from [111], 2023, ACS, available under CC BY license.

In this regard, Billah et al. synthesized chitosan–based bio-nanocomposite with multifunctional applicability by employing Layered double hydroxides (Mg/Al LDH) [111]. After characterization, the prepared nanocomposite was examined for As(V) heavy metal removal and antimicrobial performance. In fact, increased surface area as well as porosity show a significant role in microbial activity and adsorption performance. For the preparation of nanocomposite, the obtained chitin from shrimp shells was treated with NaOH to produce chitosan. Mg/Al LDH was organized in the co-precipitation process. Then, Mg/Al LDH and glutaraldehyde simultaneously were added into the chitosan solution (chitosan: LDH ratio was 3:1); then, it was stirred and treated with NaOH for precipitation. TGA of chitosan@MgAl-LDH was studied at the 25–700 °C range. An overall mass loss of 50% was seen for the TGA thermogram of chitosan@MgAl-LDH. At 200–500 °C, a 40% loss in weight was observed, attributable to the destruction of cross-linked chitosan. Above 500 °C, the polymer was destroyed, leaving behind stable oxides of magnesium and aluminum oxides. The surface area of chitosan@MgAl-LDH was 318.26 m^2^g^−1^, more than that of chitosan (96.09 m^2^g^−1^). The prepared bio-nanocomposite was exposed to up to 69.29 mgg^−1^ adsorption capacity for As(V) removal. The adsorption was spontaneous and endothermic (Δ*H*°: +10.93 kJ). It showed an 85% recovery after the seventh cycle. Additionally, bio-nanocomposite showed effective action against *Staphylococcus aureus* (inhibitory zone of 20 mm) and *Bacillus subtilis* (inhibitory zone of 21 mm) (Figure 22). Indeed, the antibacterial activity of chitosan nanocomposite is due to the polycationic structures of chitosan (electrostatic interactions among NH_3_^+^ and negatively charged cell membranes).

**Figure 23:**
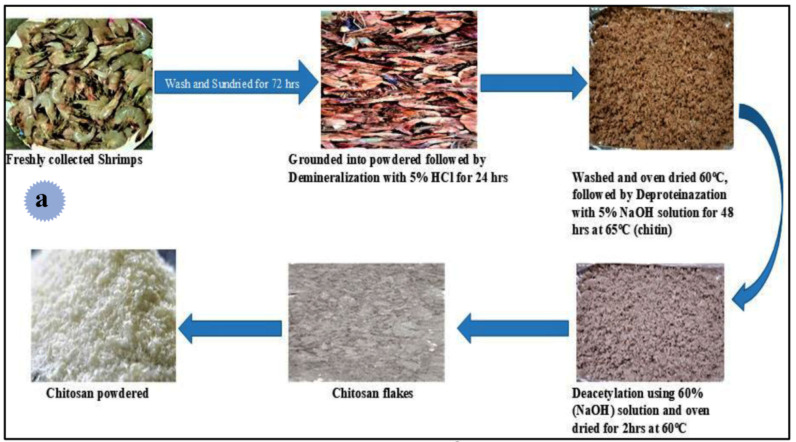

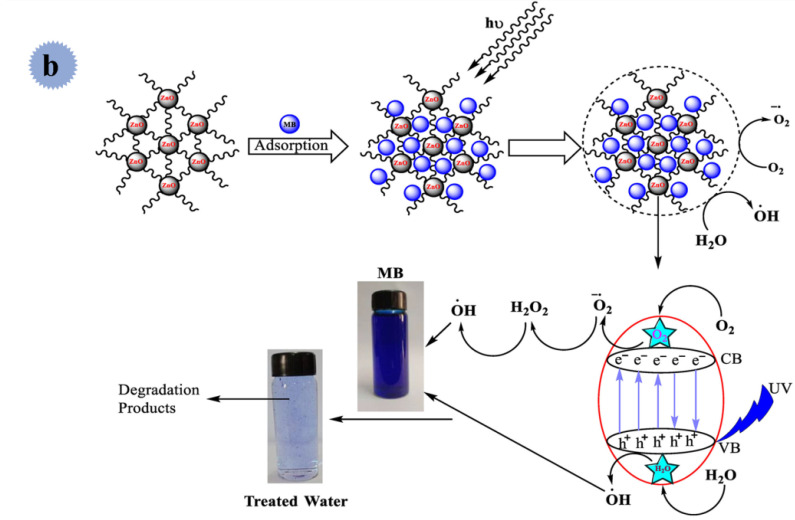
Synthesis route of chitosan nano powder from Shrimps (**a**), Synthesis route of chitosan nano powder from shrimp and (**b**) plausible mechanism depicting photodegradation of methylene blue dye. Reproduced with permission from [112], 2023, John Wiley and Sons.

In another study, Usman et al. reported the successful green preparation of ZnO/chitosan bio-nanocomposite for dye (methylene blue) photodegradation in an aqueous solution under UV irradiations [112]. In this work, shrimp were washed and dried, and their shells were grounded to form powder. They were demineralized using HCl. The prepared sample was deproteinized using NaOH at 70 °C. The chitin product was deacetylated and finally, chitosan was obtained (Figure 23a). On the other side, ZnO nanoparticles were fabricated using callistemon citrinus leaf extract. After that, chitosan solution and ZnO nanoparticle were added together and stirred at 60 °C. Precipitation was formed after adding NaOH, which was centrifuged. The fabricated nanocomposite was dried and calcinated at 500 °C. This nanocomposite showed good photodegradation performance for methylene blue (99.2% photodegradation, T = 25 °C, 120 min at pH 8) (Figure 23b). It followed pseudo-first-order kinetics and showed great reusability.

Additionally, Amirmahani et al. fabricated chitosan/ZnO bio-nanocomposite as a very effective adsorbent for reactive red 198 from water [113]. Shrimp shells were employed to prepare the extracted chitin, followed by chitosan preparation. Then, ZnO and chitosan were added to acetic acid and ultrasonicated for 30 min. The pH of the solution was adjusted to be 10. After continuously stirring (T: 40–80 °C), it was dried. The maximum adsorption capacity was 172.41 mg/g (pH: 4, 25 °C, 40 min time, and 0.1 g/L adsorbent dose).

**Table 5:** Materials, methods, and properties of chitosan nanocomposite.

Materials	Filler	Properties	Application	Ref
Sunflower oil, chitosan, and fly ash	Sunflower oil (2% (*w/v*))	Antibacterial activity by agar diffusion method could be done in two possible mechanisms: generation of ROS or electrostatic interaction among the nanocomposite and cell membrane.	Packaging	Alterary et al. [109]
Chitosan, Mg/Al LDH	Chitosan:LDH ratio was 3:1	The surface area of chitosan@MgAl-LDH was 318.26 m^2^g^−1^. The prepared bio-nanocomposite was exposed to up to 69.29 mgg^−1^ adsorption capacity for As(V) removal.	Water treatment	Billah et al. [111]
ZnO/chitosan	-	This nanocomposite showed good photodegradation performance for methylene blue (99.2% photodegradation, T = 25 °C, 120 min at pH 8)	Water treatment	Usman et al. [112]
Chitosan/ZnO	-		Water treatment	Amirmahani et al. [113]

## 5. Starch

**Figure 24:**
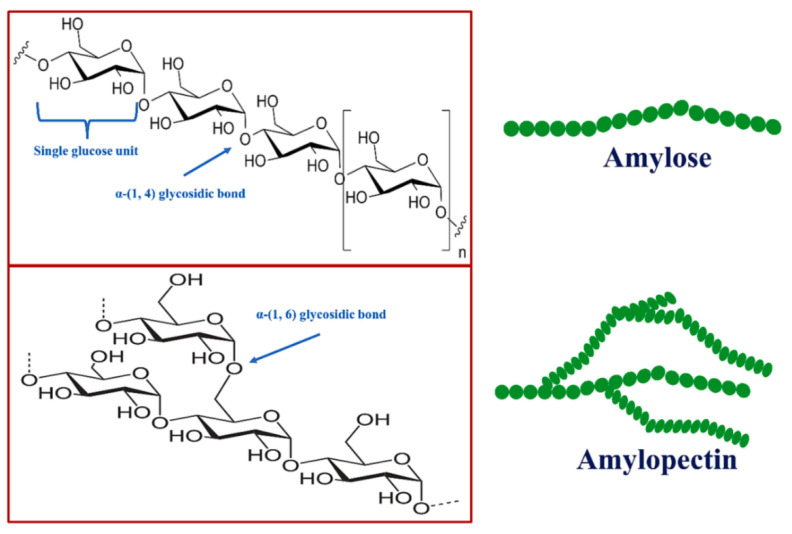
Chemical composition of starch featuring units of amylose and amylopectin. Reproduced with permission from [116], 2023, Elsevier.

As a storage polysaccharide and a major energy source expended in the diet of humans from plant sources including grains, legumes, and tubers, starch has great features, like biodegradability, abundance, renewability, low cost, and easy availability [114,115]. The utilization of starch-based products was established when Egyptians, in the predynastic period, cemented strips of papyrus together with wheat-based starch adhesive. This semi-crystalline biopolymer is a suitable candidate for film and coatings manufacture. It constitutes two components that are present in the starch granules and plays a central role in forming the structural framework: water-soluble amylose (20–30%, a linear polysaccharide composed entirely of *D*-glucose units joined by the α-1,4-glycosidic bonds) and water-insoluble amylopectin (70–80%, branched-chain polysaccharide composed of glucose units linked primarily by α-1,4-glycosidic bonds) (Figure 24) [116]. In the architecture of starch, these linear and branched molecules are formed by 1000 *α*-*D*-glucopyranosyl and 4000 glucose units [115]. Amylose–amylopectin ratios, shape and size of the starch, as well as the composition and functionality vary based on the botanical species and sources.

### 5.1. Source and Extraction Techniques of Starch

Starch is present in parts of the plants, including the seeds, fruits, or tubers. Starch can be isolated from crops, such as corn, wheat, rice, potato, and cassava. The starch extraction from potatoes is moderately simple because of the structure of the tissue and the low protein and lipid content (values below 4%). For this aim, the steps involved in extracting of potato are only milling, decantation, centrifugation, successive washes of the starch with distilled water, and finally drying. Additionally, rice grains consist mostly of starch (76–90%) and minor volumes of proteins, lipids, fibers, and ashes. The starch isolation from rice includes the employing of alkaline solvents (NaOH), surfactants, or protein-hydrolyzing enzymes for the removal of the rice protein from rice flour. To extract the protein during starch isolation, surfactants such as dodecylbenzene sulfonate and sodium lauryl sulfate are usually used. Oat is another source of starch isolation. However, the production of oat starch is difficult due to the strong bond to protein, and the presence of *β*-glucans in the grain also hinders starch separation. Generally, the isolation approaches comprise protein removal, multiple mechanical separations to eliminate cell wall debris, water washing (neutralization), and recovery through centrifugation. Wheat and corn, the main sources of cereal starches, are responsible for 99% of global starch production [117,118,119].

Sweet potato, as a very productive crop, produces 30–50% more starch than rice, maize, and wheat starch sources under similar conditions. Ghoshal et al. in 2023 studied the optimization of starch extraction from sweet potato for application in making edible films [120]. In this work, for extraction of starch, sweet potatoes were washed, peeled, and cut into pieces. After homogeneously blending them with a blender, the slurry was passed over the screen of a 100-mesh sieve and washed with DI water. Then, excess alkali solution was added and kept at 4 °C. After preparation and characterization, edible films were manufactured based on the extracted starch. The yield was in the range of 27.4–30.1 g/100 g of sweet potato starch. The ash and protein content of sweet potato starch extracted was low (0.1–0.5(%), and 0.1–0.23(%)), indicating its high purity. Many films (1:1, 2:3, 1:3, and 1:5) based on gelatine and sweet potato starch were developed, and the optimized concentration of the films. The outcomes showed a 1:5 ratio of starch and gelatin in films was the most inappropriate for packaging, and a 1:1 ratio produced better results.

**Figure 25:**
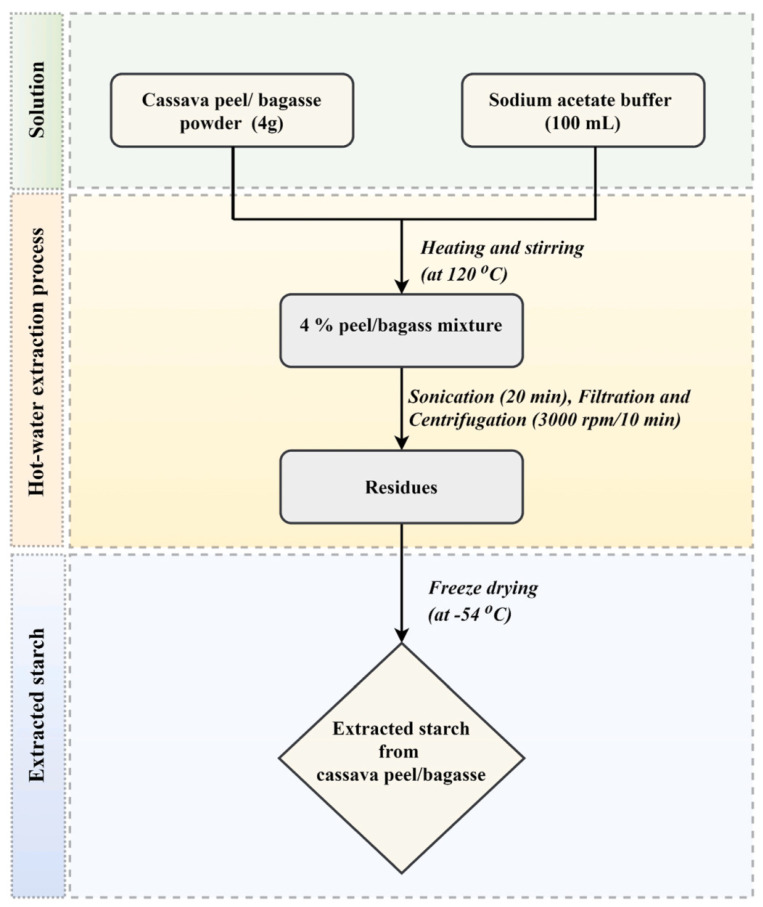
The extraction method followed to extract starch from both the cassava peel and bagasse powder. Reproduced from [121], 2023, Elsevier, available under CC BY license.

In another work, Thuppahige et al. extracted and characterized starch from cassava-industry-based waste materials (i.e., cassava peel and bagasse) using a hot-water extraction method [121] (Table 6). The obtained extracts were characterized, and their features were compared to those of commercial starch. For starch extraction, a buffer solution was prepared with sodium acetate and acetic acid in the water (pH: 4.5). After adding the cassava peel/bagasse and heating and stirring the suspension at 120 °C, it was sonicated for 20 min. The sonicated slurry was then filtered centrifuged and washed. Finally, the obtained precipitate was freeze dried, weighed, and stored in separate air-tight containers (Figure 25) Based on the outcomes, the starch yield from cassava peel was meaningfully higher than bagasse-based starch (30 ± 2% wt., and 8 ± 1% wt. respectively). Morphology and functional groups of commercial starch, cassava peels, and bagasse were very similar. Utilizing cassava peel to extract starch was more effective than that of cassava bagasse.

Mieles-Gómez et al. reported the preparation of mango (*Mangifera indica)* kernel starch via an ultrasound-assisted extraction method. In this work, diverse sonication conditions: power (120, 300, and 480 W) and time (10, 20, and 30 min) were estimated accompanied by a control treatment (extracted through wet milling technique). This reported method increased the starch yield, with the highest values (54%) at 480 W and 20 min. Ultrasound increased the yield and broke down starch chains and amylopectin, as it increased amylose values. Ultrasonically extracted starches showed an increase in solubility, amylose content, water, and oil-holding capacity, and swelling, as well as oil-holding capacity. Additionally, mango kernel starch showed antioxidant features [122]. This part is summarized in Table 6.

### 5.2. Preparation of Starch-Based Nanocomposites

Till now, different nanocomposites based on the starch have been prepared for diverse uses (Table 7). For example, recently, Arifin et al. fabricated bio-nanocomposite films based on corn starch and carboxymethyl cellulose containing different types of plasticizers (glycerol or sorbitol) incorporated with ZnO nanoparticles (0, 3, 5% wt.) via the casting method for using in the packaging sector [123]. Corn starch extraction was performed by milling corn kernels and then mixing them in water After filtering and centrifuging the resulting slurry, wet starch was dried in an oven and finally ground. ZnO and plasticizers were used to develop the bio-nanocomposite properties. The physicochemical features of the films were characterized, and the outcomes displayed that the incorporation of sorbitol could significantly enhance the value of tensile strength, flexibility, elongation, and Young’s modulus. The tensile strength of the bio-nanocomposite was in the range of 0.74 –6.07 MPa. Additionally, the elongation break was around 29.74–117.92%. After using sorbitol-plasticized film, tensile strength showed a higher value than in glycerol-plasticized film. When diverse amounts of ZnO nanoparticles were incorporated into the film, no important difference in the tensile strength value was observed. Based on the outcomes, compared to the pure film, ZnO nanoparticles in the film matrix could increase the thermal stability of the film owing to the ZnO interaction with starch. Likewise, higher concentrations of nano-ZnO (with 5 wt% ZnO) in the film increased the tensile strength, reduced the water vapor permeability, decreased the water solubility, and influenced the morphology, crystallinity, functional groups, and thermal stability of the films.

**Figure 26:**
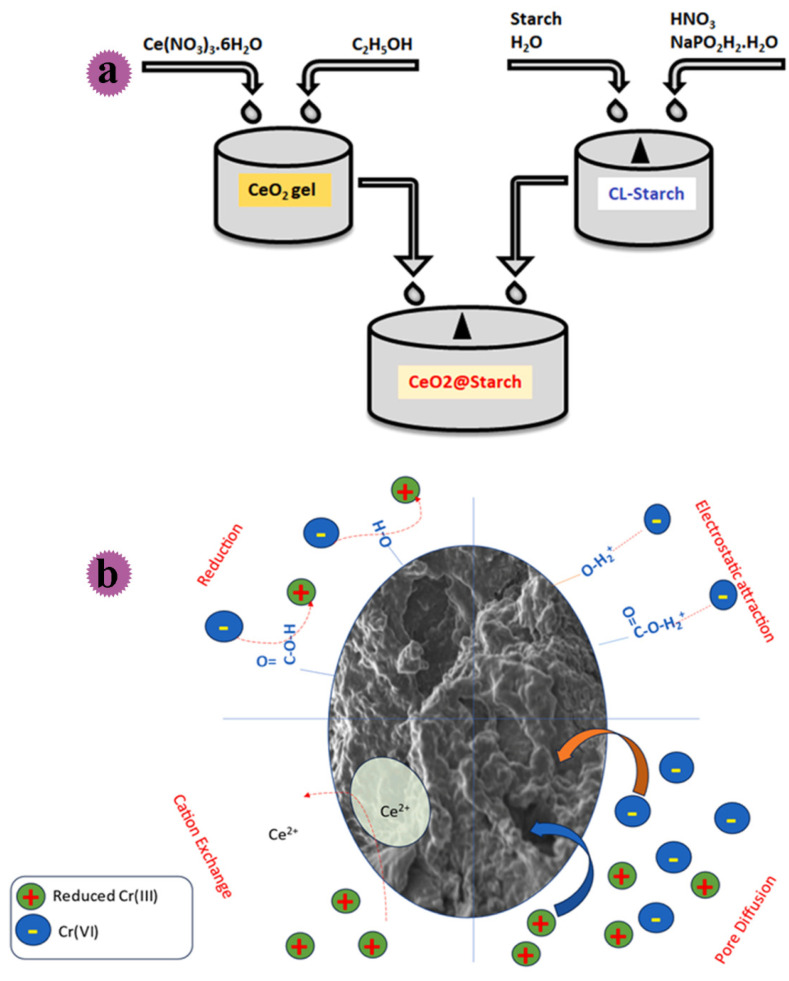
(**a**) Schematic showing the production of the nanocomposite material. (**b**) Cr(VI) and Cr(III) removal mechanisms by the CeO_2_@starch nanocomposite material. Reproduced from [124], 2023, Elsevier, available under CC BY license.

Owing to toxicity and bio-accumulative nature, heavy metal ions in industrial wastewater are an environmental problem. Cr(VI) is a dangerous pollutant due to high water solubility and oxidizing ability. Therefore, Jaiyeola and co-workers designed and characterized a very effective bio-nanocomposite of CeO_2_@starch, for the removal of Cr(VI) from aqueous solutions [124]. The production procedure of the CeO_2_@starch nanocomposite can be seen in Figure 26a. First, starch was crosslinked with a citric acid crosslinking agent in the presence of catalyst NaH_2_PO_2_ H_2_O. CeO_2_ gel was prepared by dissolving g cerium nitrate in ethanol. By combining the crosslinked starch and CeO_2_, a nanocomposite was prepared. Based on the thermo-gravimetric curve of the nanocomposite, a gradual mass loss of 10% was observed in the 25–185 °C range. CeO_2_@starch nanocomposite material didn’t show a further mass loss at temperatures above 200 °C indicating stability at higher temperatures. The prepared nanocomposite showed a remarkable capacity for the reduction of Cr(VI) ions to Cr(III) in solutions. The maximum adsorption was done at pH = 2, and equilibrium was attained in 240 min of contact time. The Langmuir isotherm model confirmed the best fit, emphasizing the monolayer adsorption. With higher temperatures, adsorption capacity increased, which showed an endothermic process. The removal mechanism of Cr(VI) from the aqueous solution can be seen in Figure 26b, the functional groups of the starch nanocomposite reduced Cr(VI) after electrostatic attachment to the surface. Additionally, Cr(III) and Cr(VI) ions, which can co-exist in solution, diffused into porous constructions of the adsorbent.

**Figure 27:**
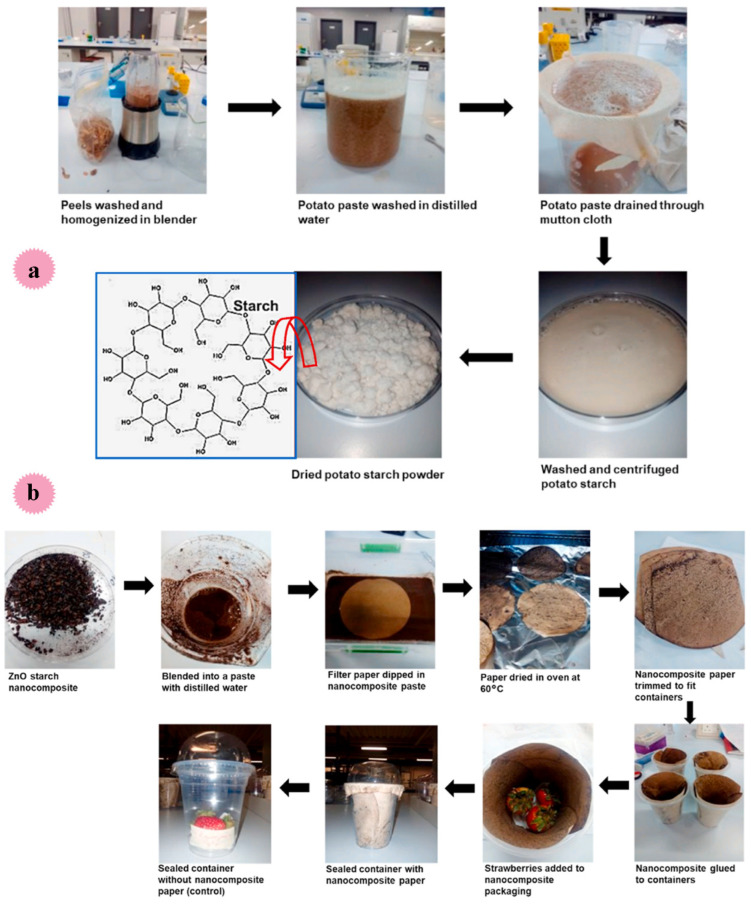
Schematic illustration of extraction of potato starch from potato peels (**a**), Synthesis of the ZnO nanocomposite paper and the development of a packaging for strawberries with ZnO-starch NC paper and without the ZnO-starch NC paper (controls) (**b**). Reproduced from [125], 2023, Elsevier, available under CC BY-NC-ND license.

In another study, Chitena et al. prepared antibacterial coatings based on ZnO/starch nanocomposite for the packaging of strawberries (*Fragaria × ananassa*). Antibacterial/antifungal features and effectiveness in prolonging the shelf life of packaged strawberries were examined [125]. For this aim, at first, a paste of potato peels was prepared using a blender and distilled water. After washing and centrifuging, the starch was obtained (Figure 27). Then, the hydrothermal technique was applied to prepare ZnO nanoparticles with hexagonal wurtzite structure and 30–40 nm size. Amounts of 10%, 15%, 18%, and 20% *w*/*w* zinc to starch were used for the ZnO/starch nanocomposites. The antifungal (*B. cinerea*) and antibacterial (*E. coli*, *P. aeruginosa*, *S. aureus* and *B. subtilis*) activities of the ZnO/starch paper were determined by the spread plate methods. Compared to the control sample (untreated fruits), ZnO/starch nanocomposite paper enhanced the shelf life of the fruits during incubation at 4 °C and resulted in fruits with acceptable quality.

## 6. Cellulose

**Figure 28:**
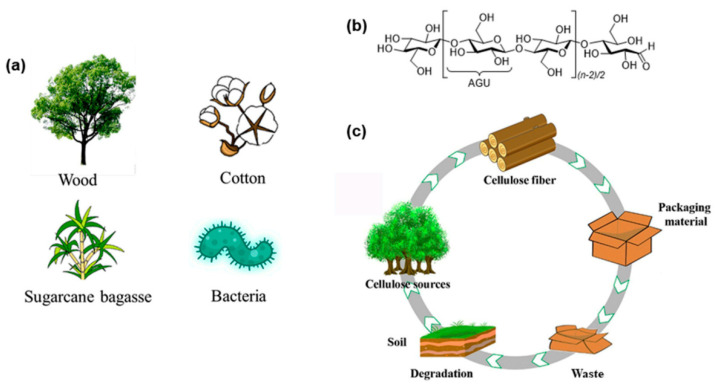
(**a**) Many common sources of cellulose; (**b**) chemical structure of cellulose; (**c**) life cycle of cellulosic food packaging. Reproduced from [126], 2022, MDPI, available under CC BY license.

Cellulose is the most abundant biopolymer present on earth. Independently of its source, it is a tasteless, colorless, semicrystalline polysaccharide and a linear biopolymer composed of repeated *b*-1,4-linked D-anhydroglucose units (AGU) with three hydroxyl groups (–OHs) per unit (Figure 28b) [126]. It is non-miscible in aqueous solvents and other organic ones. The deprived solubility is attributed primarily to the strong intramolecular and intermolecular -H bonding holding the individual chains [127]. In fact, the highly ordered anisotropic arrangement and strong intra- and inter-chain interactions make cellulose fibers with high axial strength and stiffness. Compared to many other polysaccharides like starch and dextrin which are amorphous, cellulose is a “Semi-crystalline” polysaccharide. The existence of hydroxyl functional groups on it results in the interlinking of the chains by H-bonding, due to which it gains fibrous properties and high tensile strength as well. Compared to conventional petroleum-based polymers, cellulosic materials show higher thermal stability, improved mechanical strength, better application qualities, and film-forming capability [126]. In 1838, Anselme Payen as a French chemist obtained this white biomacromolecule for the first time and named this new substance “cellulose” because he had obtained it from the cell walls of plants. In 1920, Herman Staudinger recognized its chemical construction. In 1992, Kobayashi and Shoda first chemically synthesized the compound. The annual construction of this biopolymer is estimated to be between 10^10^ and 10^11^ t. However, a small portion of 6 × 10^9^ t is exploited by a number of industrial arenas like textile, paper, and chemicals in addition to material industries. Cellulose has several potential uses in diverse industries, including composites, packaging, straws, flexible electronics, tissue engineering materials netting, upholstery, coatings, and paper, so, researchers paid attention to extracting cellulose from plants because cellulose is a renewable resource because of its biodegradability and biocompatibility. The advantages of natural cellulose fibers are biodegradability, nontoxicity, cheapness, high specific strength, and eco-friendly content [126,128,129].

### 6.1. Source and Extraction Techniques of Cellulose

Cellulose is isolated with a number of natural fibers, like plant biomass (wood, agricultural residues, and dedicated energy crops), bacteria (like *Gluconacetobacter, Acetobacter, and Komagataeibacter*), algae (such as *Cladophora* and *Ulva*), insects, and minerals. Biomass—for example, plant resources or agro waste—is the main source of cellulosic fiber [128,130,131]. Extraction of cellulose can be attained in nano or microforms via chemical or mechanical methods [130]. Extraction of cellulosic fiber is commonly performed in three major steps: pre-hydrolysis treatment that is performed by mineral acid or alkali so as to open up the matrix. Pre-treatment, that is to eliminate or alter components that impede the crystallization process, such as lignin, is considered a crucial step in the production of crystalline cellulose from different sources. Pre-treatment can be performed by chemical, physical, and biological processes. Pulping is to cook the fiber using an alkali, like NaOH. Bleaching, the final step, extracts the pure bleached form of cellulose and can be performed with many materials, like H_2_O_2_. To raise the purity of the obtained cellulose, hydrolysis with sulfuric acid in a reflux medium is carried out [127].

In 2023, many investigations have been conducted for cellulose extraction from different sources (Table 8). For example, Abzan and coworkers reported the cellulose microfiber extraction from leftover celery pulp [132]. In this work, two chemo-mechanical treatments counting bleaching with NaClO_2_ and NaOCl for pure cellulose extraction from leftover celery pulp (Apium graveolens var. dulce). Optical microscopy and morphology investigations indicated that the diameters of the untreated cellulose fibers ranged from 100 to 150 μm, and for the treated ones, they ranged from 10 to 15 μm. Additionally, the outcomes showed the high stabilities of products in thermal and mechanical conditions. Chen et al. reported the extraction of cellulose nanocrystals from hardwood pulp [133]. Two kinds of cellulose nanocrystals were obtained through the centrifugation of the reaction suspension after the ammonium persulfate oxidation of these cellulose fibers. Dependent on ultrasonication, the cellulose nanocrystals were classified as 1 and 2. Cellulose Nanocrystal 1 showed a higher surface charge, higher crystallinity, higher thermal stability, shorter length, smaller length distribution, and slightly larger width. The maximum yields were 34.11% and 42.65%. Freixo et al. used sugarcane bagasse (major by-product of the sugarcane industry) as feedstock to produce cellulose or cellulose-based materials [134]. To obtain more purified cellulose, pretreatment procedures of lignocellulosic feedstocks were used for the composition of the complex cellulose–ligninhemicellulose matrix. In fact, autohydrolysis was used as a cost-effectiveness fractionation method owing to the excessive selectivity in extracting hemicellulose. In this process, hot water (usually ranging from 160 to 240 °C) and 10–35 bar pressure were used without the chemical agents. The effect of many conditions, alkaline sulfite extraction, and bleaching were evaluated. Autohydrolysis at 170 °C followed by a bleaching step was the treatment that presented the best results in terms of cellulose purity (77.8%) and crystallinity (73.4%).

### 6.2. Preparation of Nanocomposites Based on Cellulose

Numerous bio-nanocomposites based on cellulose have been developed for many applications, especially photocatalysts (Table 9) [135]. For example, cellulose has been widely used in supporting and dispersing ZnO catalysts. In this regard, Shi et al. prepared ZnO/cellulose nanocomposites by growing ZnO sheets in situ on cellulose nanofibers [136]. For this aim, cellulose nanofibers were prepared and added into the ZnCl_2_ aqueous solution. After stirring the mixture (speed of 200 rpm to mix well), NaOH aqueous solution was poured into it, followed by stirring to attain ZnO/cellulose nanocomposites. The outcomes showed that the diameter, sheet number, as well as thickness of flower-like ZnO in porous nanocomposites were improved by the enhancement of cellulose surface electronegativity. The fabricated bio-nanocomposite with surface carboxyl content of 1.63 mmolg^−1^ and zeta potential of –52.6 mV had the highest photocatalytic activities for dye degradation owing to the strong electrostatic adsorption between carboxyl and ZnO as well as exposure of the interfacial active sites.

**Figure 29:**
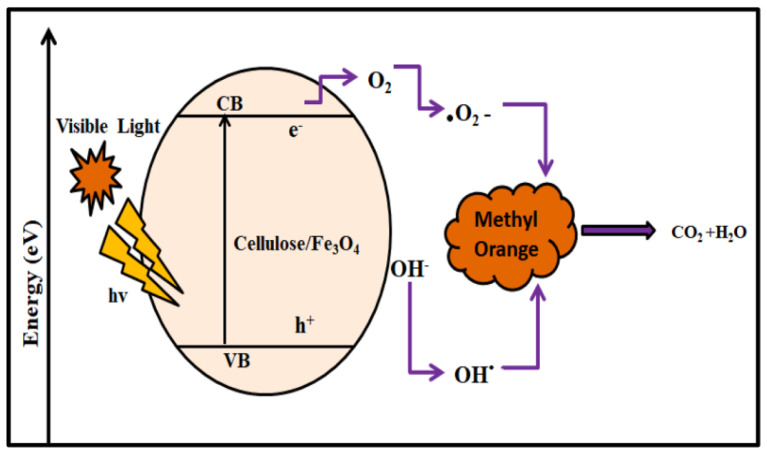
Photocatalytic mechanism of nanocomposite of cellulose/Fe_3_O_4_ nanocomposite. Reproduced with permission from [137], 2023, Elsevier.

In another work, Arularasu et al. biosynthesized cellulose/Fe_3_O_4_ nanocomposite that this nanocomposite was prepared from *Kappaphycus alvarezii* plant extract in an easy technique via the co-precipitation method. A 1:1 ratio of cellulose and Fe_3_O_4_ was employed. The cellulose matrix had many roles, such as a capping agent and stabilizing agent as well as support against accumulation of nanoparticles. Bio-compatibility and photocatalytic activity (for methyl orange) of the nanocomposite were examined [137]. FE-SEM descriptions exposed rods and spherical shapes of cellulose/Fe_3_O_4_ nanocomposites with 20–30 nm particle sizes. This nanocomposite with a 2.79 eV bandgap depicted the best active antibacterial and photocatalyst. The cellulose nanocomposite displayed great antibacterial and antioxidant performance compared with Fe_3_O_4_ nanoparticles. It showed (17.3 ± 0.5 mm) at 2.5 µg/mL against *E. coli* (MTCC 443) and a minimum inhibition zone of (9.6 ± 1.1 mm) on *Staphylococcus aureus* (MTTC 3615) at 0.5 µg/mL, respectively. It showed high dye degradation efficiency (96.25%) at 120 min. Based on Figure 29, by absorbing the visible light on the surface of the nanocomposite, electrons/holes are formed via transferring electrons from the valence band to the conduction band. The electron and holes in bands create peroxide radical anions (•O_2_) and hydroxyl radicals (•OH) to break dye to CO_2_ and H_2_O.

Additionally, Hong et al. reported the production of a novel construction of cellulose-TiO_2_ nanocomposite as a photocatalyst for dye degradation in wastewater. Non-sintering, low-temperature sol–gel, and co-precipitation methods were used to prepare the nanocomposite. The fabricated cellulosic nanocomposite revealed 100% removal of dye in 30 s under diverse temperatures and pH conditions. The removal rate of dye remained at 72% after 7 times of duty-cycle operation [138].

## 7. Conclusions and Prospects

Polysaccharides are considered a sustainable and green alternative to conventional petrochemical-based polymers. These materials originate from plants, animals, seaweed, mushrooms, and microorganisms. Nevertheless, many inherent features of these polymers like poor mechanical and hydrophilicity limit their utility as a direct replacement of conventional material. Thanks to nanotechnology, fabrication of materials at the nanoscale significantly develops the applicability and recombination of these materials with biopolymers, resulting in bio-nanocomposites. In fact, the technological innovations in nano and polymer technology have paved the way for developing bio-nanocomposites and their exploration in diverse fields. In this review, many common polysaccharides—specifically, alginate, pectin, chitosan, starch, and cellulose—were introduced, and properties and applications of them were expressed. In the next step, extraction methods for the biopolymers from different sources and different methods were stated. After that, the fabricated bio-nanocomposites based on biopolymers for diverse applications were introduced. Regarding alginate, films made from this biopolymer along with chitosan and quantum dots are visible-light-responsive and possess improved mechanical and thermal stability as well as antibacterial properties, making them practical for preserving fruits like kiwifruit. Furthermore, alginate microgel beads containing Ag nanoparticles have exhibited strong antibacterial and anticancer effects. Additionally, nanocomposites of alginate and metal oxides have shown effectiveness in removing various pollutants, such as heavy metals and dyes, improving their efficiency in water treatment. Pectin-based nanocomposites have shown great potential for active and edible food packaging applications. Indeed, these materials can control the migration of gases, moisture, and lipids in packaging. However, the barrier and antimicrobial properties of pectin films are often inadequate, so incorporating antimicrobial agents such as essential oils, nanotubes, metal oxides, and graphene oxide can enhance their functionality in the food industry and medicinal applications. Additionally, chitosan-based nanocomposites have attracted considerable interest due to their versatile properties. Studies indicate that adding fillers such as sunflower oil, double metal hydroxides, and metal oxides to the chitosan matrixes, enhances their functionality for various applications across multiple industries, including food processing, and wastewater treatment. On the other side, starch-based nanocomposites have been developed for various applications, particularly in packaging and water treatment. For instance, the prepared CeO_2_@starch nanocomposite shows a remarkable capacity for the reduction of Cr(VI) ions to Cr(III) in solutions. In food packaging, research shows ZnO/starch nanocomposite demonstrated significant antibacterial and antifungal properties. Besides, fabricated cellulose-based nanocomposites have a wide range of applications, especially in photocatalysis and biomedical fields. Nanocomposites incorporating cellulose with ZnO, Fe_3_O_4_, and TiO_2_ have demonstrated impressive photocatalytic abilities for removing dyes, achieving efficiencies of up to 100% removal within a short time under ideal conditions. Therefore, based on the outcomes the developed nanocomposites have diverse applications like food, water treatment, biomedical, and so on. Nevertheless, the possibility of the noxious influence of nanofillers in nanocomposites on human health and the environment should not be ignored. For example, the addition of nanoparticles, like Ag can advance the mechanical and antimicrobial features of food active packaging. However, these nanoparticles may hurt human health. This has led to legislative and regulatory worries. Packaging comprising particles can decrease waste by improving the shelf life of products. Although the packaging process can be a source of chemical contamination of foods. Therefore, the migration of nanoparticles from bio-nanocomposite in the packaging of the food product is one of the challenging aspects, consequently, it should be taken into consideration. Estimate of toxicity effect on human health and environment is another area. After the recent COVID-19 pandemic, the development of more antiviral materials could be a new area worth speeding up. On the other side, the biodegradability of bio-nanocomposites is one of the main concerns for environmentalists and institutions governing public health safety. Indeed, the biodegradability of bio-nanocomposites should be studied for several key reasons: 1. Environmental impact: Bio-nanocomposites are often designed as eco-friendly alternatives to traditional materials. Studying their biodegradability ensures that they break down in the environment without leaving harmful residues, helping to reduce pollution and waste accumulation. 2. Sustainability: Understanding the biodegradation process allows for optimizing the composition of bio-nanocomposites to enhance their sustainability. It helps in developing materials that can be used in various industries without negatively affecting ecosystems when they are discarded. 3. Life cycle assessment: Evaluating the biodegradability of these materials provides essential data for the complete life cycle assessment of products. These data help in assessing their true environmental benefits compared to synthetic counterparts. 4. Waste management: The study of biodegradability informs waste management strategies, helping policymakers and manufacturers create products that fit into existing or improved composting and recycling systems. 5. Regulatory and commercial viability: With the growing demand for eco-friendly products, regulations are becoming stricter on material sustainability. Understanding biodegradability helps companies meet regulatory requirements and market their products as environmentally responsible. In summary, studying the biodegradability of bio-nanocomposites is essential to ensuring that these materials contribute to sustainability without causing unintended harm to the environment. Therefore, researchers should further investigate this issue.

## Figures and Tables

**Table 1:** Materials, methods, and properties of extracted alginate.

Source	Extraction Method	Yield	Properties	Ref
Different European Brown algae species:*Saccharina latissima* (SAC), *Laminaria digitata* (LAM), *Sacchoriza polyschides* (SACC), and *Himanthalia* spp. (HIM)	Sustainable protocol based on Citric acid	In small scale: 61–65% for SAC and LAM, and 34–41% for SACC and HIM. Scaling-up extraction: 26–30% (30% for SAC, 37% for LAM, 32% for HIM and 26% for SACC).	The ManA/GulA ratios were 1.98 for SAC, 2.23 for LAM, 1.83 for SACC, and 1.86 for HIM and similar to the value obtained for the commercial alginate 1.84).	Silva et al. [58]
*Alaria esculenta* and *Saccharina latissima*	Alkaline extraction	At optimum condition (pH 9), had a yield of 185 ± 7 and 229 ± 12 mg/g dry-weight seaweed	The purity of the extracted alginates was evaluated based on the content of coextracted impurities and was found to be comparable with high-quality commercial alginates.	Nøkling-Eide et al. [59,60]
Brown seaweeds (*Padina boergesenii*, *Turbinaria triquetra*, *Hormophysa cuneiformis*, *Dictyota ciliolata*, and *Sargassum aquifolium)*	Alkaline extraction	The highest yield of was from *T. triquetra* (22.2 *±* 0.56% DW), while the lowest content was observed in *H. cuneiformis* (13.3 *±* 0.52% DW).	The alginate extracted from the five selected seaweeds also demonstrated food-grade quality.	Rashedy et al. [61]

**Table 2:** Materials, methods, and properties of alginate nanocomposite.

Materials	Filler	Properties	Application	Ref
Na-alginate, chitosan, quantum dots, and metal-organic frameworks (ZIF-8) layer-by-layer (LBL) assembly method	Amount of 0.1%, 0.25%, and 0.5%	Good antibacterial performance (>99%) against foodborne microorganisms under visible light irradiation and >40% ethylene scavenging feature.	Packaging	Wang et al. [62]
Ag nanoparticles, alginate	-	Promising antibacterial and anticancer properties.	Medical	Hou et al. [63]
Na-alginate/black nano TiO_2_	Amount of 0, 0.02, 0.2, and 2% (*w/v*)	Hydrogel containing 2% (*w/v*) TiO_2_ showed the highest dye degradation potential (99% of both methylene blue (by 180 min) and malachite green (by 360 min)).	Water treatment	Kumar et al. [64]
Alginate-g-polyallylamine/reduced graphene oxide/CuO	-	In Cd^2+^ sensing, the limit of quantitation and limit of detection were 4.24 nM and 1.27 nM, respectively. This sensor was stable, and reproducibility was satisfactory.	Water treatment	Tripathy et al. [65]

**Table 3:** Materials, methods, and properties of pectin nanocomposite.

Materials	Filler	Properties	Application	Ref
Halloysite nanotubes, grapefruit seed oil, pectin	Different amounts of grapefruit seed oil 20, 30, and 50% *w*/*w*)	Increase in water permeability up to 480%, 200%, increase in elastic modulus, and deformation at breaking up to 39%.	Packaging	Viscusi et al. [79]
Pectin, poly (vinyl alcohol), MgO	Amount of 0.5 g of pectin and 0.15 g of poly (vinyl alcohol) 2.4 g of Mg(NO_3_)_2_⋅6H_2_O	Bio-nanocomposite film with MgO displayed increased antioxidant activity, low solubility, and decreased water vapor permeability.	Packaging	Suhasini et al. [80]
Pectin, cellulose nanocrystals, montmorillonite nanoparticles, and chitosan	Amount of 2.5 wt.% of montmorillonite and cellulose	All pectin-based films showed higher oxygen barrier features than polyethylene.	Packaging	Souza et al. [81]
Pectin, ZnO	ZnO nanoparticles (5w/w% relative to pectin),	Bio-nanocomposites presented good antimicrobial and antioxidant. Activity.	Food packaging	Przybyszewska et al. [82]
Pectin, pracaxi oil	Pracaxi oil at 0, 0.1, 0.2, 0.3, and 0.4% wt	The antioxidant oil with the presence of phenolic compounds efficiently enhanced the butter stability against oxidation processes during the 60 days of storage.	Food packaging	Candido et al. [83]
Pectin/lignocellulose/chitin	Amount of 52%, 31%, and 17%, respectively in 1% (*w/v*)	The maximum adsorption capacity was 5578.4 mg/g bile salts and 37.9 mg/g cholesterol.	Medicine and food industry	Khorasani et al. [84]
Pectin, Fe_3_O_4_	-	The product as an antihuman colorectal carcinoma bio-nanocomposite exhibited high antioxidant activity toward DPPH	Medicine	Wang et al. [85]
Apple pectin, arabinoxylan, hydroxyapatite, and graphene oxide	Amount of 0.1, 0.2, 0.3, and 0.4 mg of graphene oxide	Graphene oxide improved physicochemical as well as biomechanical features of composite.	Medicine	Al-Arjan et al. [86]

**Table 4:** Materials, methods, and properties of extracted chitosan.

Source	Extraction Method	Yield	Properties	Ref
Shrimp shells	Chemical extraction by NaOH, HCl	-	Chitosan with 65% deacetylation degree and with rod-like micro-structure was effectively produced.	Hisham et al. [106]
Shrimp shells	Enzymatic methods	Maximum demineralization values were 98.64 and 97.57% for lactic 15 and acetic acids, respectively.	Physicochemical analysis indicated that the enzyme assisted production of chitin seems appropriate to extract chitin, possibly retaining its native structure.	Hongkulsup et al. [107]
Seafood waste	Microbial extraction	Potato peel had the highest dry weight of chitin (0.89 g yield).	Red grape pomace resulted in 72.90% deacetylation degree and 95.54% crystallinity degree.	Nian Tan et al. [108]

**Table 6:** Materials, methods, and properties of extracted starch.

Material	Method	Yield	Properties	Ref
Sweet potato, *gelatine*	Alkali extraction	27.4–30.1	The ash and protein contents were low (0.1–0.5(%), and 0.1–0.23(%)), which showed high purity of the product.	Ghoshal et al. [120]
Cassava (*Manihot esculenta*) agro-industrial wastes	Hot-water extraction method	30 ± 2% wt	Employing cassava peel to extract starch was more effective than that of cassava bagasse.	Thuppahige et al. [121]
Mango (Mangifera indica) kernel	Ultrasound-assisted extraction	54%	A significant increase in the amylose content, water-holding capacity, oil-holding capacity, solubility, and swelling power of ultrasonically extracted starches was observed.	Mieles-Gómez et al. [122]

**Table 7:** Materials, methods, and properties of starch nanocomposite.

Materials	Filler	Properties	Application	Ref
Corn starch, carboxymethyl cellulose, ZnO nanoparticles	0, 3, 5 wt.%	Higher concentrations of nano-ZnO (with 5 wt% ZnO) in the film increased the tensile strength, reduced the water vapor permeability, decreased the water solubility, and influenced the morphology, crystallinity, functional groups, and thermal stability of the films.	Packaging	Arifin et al. [123]
CeO_2_@starch	-	The maximum adsorption was done at pH = 2, and equilibrium was attained in 240 min of contact time.	Water treatment	Jaiyeola et al. [124]
ZnO/starch	10%, 15%, 18%, and 20% *w*/*w*	Compared to the control sample (untreated fruits), ZnO/starch nanocomposite paper enhanced the shelf life of the fruits during incubation at 4 °C and resulted in fruits with acceptable quality.	packaging	Chitena et al. [125]

**Table 8:** Materials, methods, and properties of extracted cellulose.

Materials	Method	Properties	Yield	Ref
Leftover celery pulp (*Apium graveolens* var. *dulce*)	Chemo-mechanical procedures including bleaching with NaClO_2_ and NaClO	Fibers with 100 to 150 μm, improvement in the mechanical and thermal stabilities for treated fibers	-	Abzan et al. [132]
Hardwood pulp	Ammonium persulfate oxidation	The surface charges: −33.6 mV to −44.5. Crystallinity indexes were 80.07 and 75.42 %. The lengths of the crystals were 157.31 ± 30.61 and 214.92 ± 65.52 nm.	34–42%	Chen et al. [133]
Sugarcane bagasse	Autohydrolysis and alkaline sulfite hydrothermal approaches, followed by bleaching treatments,	Autohydrolysis at 170 °C for 1 h followed by a bleaching step was the treatment that presented the best results in terms of cellulose purity (77.8%), crystallinity (73.4%).	Autohydrolysis at 170 °C yielded 63.5% of a solid fraction with 69.4 ± 0.4% of cellulose, while alkaline sulfite at 180 °C yielded 50.9% of a solid fraction with 72.1 ± 5.8% cellulose.	Freixo et al. [134]

**Table 9:** Materials, methods, and properties of cellulose nanocomposite.

Materials	Filler	Properties	Application	Ref
ZnO/cellulose	1 g of cellulose and 50 mL 0.2 M ZnCl_2_	It had the highest photocatalytic activities for dye degradation owing to the strong electrostatic adsorption between carboxyl and ZnO as well as exposure of the interfacial active sites.	Photocatalyst	Shi et al. [136]
Cellulose/Fe_3_O_4_	1:1 ratio of cellulose and Fe_3_O_4_	It showed high dye degradation efficiency (96.25%) at 120 min.	Photocatalyst	Arularasu et al. [137]
Cellulose-TiO_2_	-	100% removal of dye in 30 s under diverse temperatures and pH conditions.	Photocatalyst	Hong et al. [138]

## Data Availability

Not applicable.
